# Extension of partial atom-to-atom maps: uniqueness and algorithms

**DOI:** 10.1186/s13015-026-00300-5

**Published:** 2026-06-19

**Authors:** Marcos E. González Laffitte, Tieu-Long Phan, Peter F. Stadler

**Affiliations:** 1 Center for Scalable Data Analytics and Artificial Intelligence Dresden-Leipzig (ScaDS.AI), Leipzig University, Humboldtstrasse 25, 04105 Leipzig, Saxony Germany; 2 Bioinformatics Group, Department of Computer Science and Interdisciplinary Center of Bioinformatics, Leipzig University, Haertelstrasse 16-18, 04107 Leipzig, Saxony Germany; 3 Department of Mathematics and Computer Science, University of Southern Denmark, Campusvej 55, 5230 Funen, Odense, Denmark; 4 Max Planck Institute for Mathematics in the Sciences, Inselstrasse 22, 04103 Leipzig, Saxony Germany; 5 Institute for Theoretical Chemistry, University of Vienna, Waehringer Strasse 17, 1090 Vienna, Austria; 6 Facultad de Ciencias, Universidad National de Colombia, Carrera 45, 111321 Bogotá, Cundinamarca Colombia; 7 Santa Fe Institute, 1399 Hyde Park Rd, Santa Fe, New Mexico 87501 USA

**Keywords:** Atom-to-atom maps, Imaginary transition state (ITS), Condensed graph of the reaction (CGR), Chemical reaction mechanisms, Molecular graphs, Metabolic networks, Chemical synthesis planning, Constrained graph isomorphism

## Abstract

Chemical reaction databases typically report the molecular structures of reactant and product compounds, as well as their stoichiometry, but lack information, in particular, on the correspondence of reactant and product atoms. These *atom-to-atom maps* (AAM), however, are crucial for applications including chemical synthesis planning in organic chemistry and the analysis of isotope labeling experiments in modern metabolomics. AAMs therefore need to be reconstructed computationally. This situation is aggravated, furthermore, by the fact that chemically correct AAMs are, fundamentally, determined by quantum-mechanical phenomena and thus cannot be reliably computed by solving graph-theoretical optimization problems defined by the reactant and product structures. A viable solution for this problem is to shift the focus into first identifying a *partial* AAM containing the reaction center, i.e., covering the atoms incident with all bonds that change during a reaction. This then leads to the problem of extending the partial map to the full reaction. The AAM of a reaction is faithfully represented by the *Imaginary Transition State* (ITS) graph, providing a convenient graph-theoretic framework to address the questions of when and how a partial AAM can be extended. We show that an unique extension exists whenever, and only if, these partial AAMs cover the reaction center. Moreover, uniqueness results are generalized to partial AAMs in situations where hydrogen atoms are not represented explicitly. In this case their extension can be computed by solving a constrained graph-isomorphism search between specific subgraphs of ITS graphs. We close by benchmarking different tools for this task.

## Introduction and motivation

A large part of chemical knowledge is encoded in chemical reactions and formalized as transformations of structural formulae, i.e., of labeled graphs that explicitly represent atoms as vertices and chemical bonds as edges. Large-scale data bases, such as Reaxys^®^[2] or the United States Patent and Trademark Office (USPTO) database [3], collect this knowledge in the form of some model for a reaction $G \longrightarrow H$, where *G* and *H* are the disjoint unions of the structural formulae of reactants and products, respectively. The same representation is used for metabolic reactions in the KEGG and EcoCyc databases.

For a wide variety of practical applications, ranging from chemical synthesis planning to the analysis of isotope labeling experiments in metabolic networks, the knowledge of *G* and *H* is insufficient, however. In addition, the exact correspondence of reactant and product atoms is required. This *atom-to-atom map* (AAM) is usually represented as a bijection $\alpha : V(G) \rightarrow V(H)$ between vertex sets of the reactants-graph *G* and the products-graph *H* modeling $G \longrightarrow H$. The AAM, moreover, preserves atom types and determines the bonds that are formed, broken and conserved in the course of the reaction [4]. Thus, AAMs can also be understood as a summary of the mechanism of a reaction, at least at the level of abstraction defined specified by structure formulas.

The databases introduced above, for example, typically do not provide AAMs together with the reactions, which therefore need to be constructed by computational means. This has turned out to be a non-trivial problem that still remains an area of active research. The main difficulty is that the *chemically correct* AAM is determined by the ground-truth mechanism of the reaction (or reactions in the case of multi-step transformations which are of particular relevance in enzyme-catalyzed biochemical reactions), which is inherently a quantum-mechanical phenomenon whose outcome is, at best, approximated by heuristic rules such as the Principle of Minimal Chemical Distance [5], geometric rules such as the Principle of Least Nuclear Motion [6], or maximum common subgraph approaches [7]. More recently, machine-learning methods have been devised to complement the shortcomings of the combinatorial methods, see [8] for are recent comparative benchmarking effort.

An alternative for seeking to infer an AAM in a single step, is to divide this task into three potentially easier subproblems: (a) determine the most likely *type* of the reaction, (b) identify the reaction center(s), i.e., the atoms incident with all bonds that change, and (c) construct the AAMs using the results of (a) and (b) as constraints.

The rationale for this approach is that, fundamentally, reactions are not arbitrary changes of bonds. On the contrary, chemical reactions usually follow specific localized patterns of bond changes and are restricted to particular classes of reactants. In the chemistry literature, such reaction types are often referred to as “name(d) reactions” such as Grignard reaction, Claisen condensation or Friedel–Crafts acylation [9].

Reaction types can be specified, moreover, by *reaction templates* describing the parts of the involved molecules that are actually affected and/or necessary for the reaction to take place. A reaction template thus is a pair of pattern graphs, one, *L* in the reactants-graph *G* and the other, *R*, in the products-graph *H*, together with a bijection $\xi : V(L) \rightarrow V(R)$ that establishes the AAM at the level of the *patterns*. A reaction template $L \longrightarrow R$ is said to *explain* the reaction $G\longrightarrow H$ if (i) *L* and *R* appear as (are isomorphic to) subgraphs of *G* and *H*, respectively, (ii) the bijection $\xi$ can be extended to an AAM for *G* and *H*, and (iii) all bonds that change during $G \longrightarrow H$ are covered by $L\longrightarrow R$. Reaction templates therefore contain and typically extend beyond the reaction center. Formally, they can be interpreted as a special case of double pushout graph rewriting rules, see e.g. [10–12]. Preservation of mass and atom types, i.e., of the number of vertices and their labels, makes it possible to express the necessary theory in terms of graph isomorphisms and induced subgraphs. We therefore forego a description of the category-theoretic formalism of graph transformations for the purposes of this contribution.

### Definition 1

Consider two reactions $G \longrightarrow H$ and $L \longrightarrow R$, where the latter is endowed with an AAM $\xi : V(L) \rightarrow V(R)$. Then $(L, R, \xi )$ is a *pattern* for $G \longrightarrow H$, if there are subgraph isomorphisms $\mu : V(L) \rightarrow V(G)$ and $\nu : V(R) \rightarrow V(H)$ of the patterns into reactants and products, such that the induced *partial atom-to-atom map* $\pi :\mu (L)\rightarrow \nu (R)$ given by $\pi (x) = \nu (\xi (\mu ^{-1}(x)))$ for all $x\in \mu (L)\subseteq V(G)$, can be extended to an AAM $\alpha : V(G) \rightarrow V(H)$ that coincides with $\pi$ on $\mu (L)$, i.e., $\alpha (x) = \pi (x)$ for all $x\in \mu (L)$.

Throughout this contribution we will assume that a reaction $G \longrightarrow H$ and a partial AAM $\pi : U \rightarrow W$ with $U \subseteq V(G)$ and $W \subseteq V(H)$ are given. In practice, such partial AAMs can be produced, e.g.  by learning-based reaction mapping tools [13, 14]. Another source of partial AAM data are the RCLASS data provided by the KEGG database, although in this case extensive processing is required to obtain the partial AAMs explicitly [15]. We therefore do not need to concern ourselves with pattern graphs *L* and *R* and their embeddings $\mu$ and $\nu$ into the graphs *G* and *H*. The mathematical structure of full and partial AAMs has been studied in some details in [4, 16]. A key observation is that AAMs over balanced reactions can be represented equivalently by means of *Imaginary Transition State (ITS)* graph and its subgraphs. ITS graphs were introduced by Shinsaku Fujita [17] and Wilcox and Levinson [18] for storage and processing of reactions in chemical databases, and later utilized under the name Condensed Graph of the Reaction (CGR) in machine learning applications [19]. The one-to-one correspondence between AAMs and ITS graphs is, therefore, the basis for the graph-theoretical approach to AAMs that we pursue in this contribution.

**Fig. 1:**
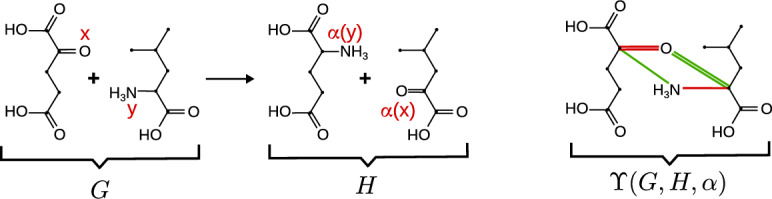
The reaction mechanism imposed by the AAM $\alpha$ over $G \longrightarrow H$ is characterized by the ITS graph $\Upsilon (G, H, \alpha )$, whose labels preserve all the information about the changing and conserved bonds

Applications to Bioinformatics of our algorithms, therefore, are strongly dependent on their applications to Organic Chemistry. Figure 1, for example, exhibits the biochemical exchange mechanism, and the corresponding ITS graph, that allows *E. coli* to recycle and route nitrogen in an efficient way [20]. Studying domain-specific mechanisms such as this one is the focus of separate intended future contributions. In order to elucidate the mechanisms of such reactions, nonetheless, one requires in the first place to address the problem of comparing and extending the associated AAMs in a mathematically correct fashion. Here we will focus in particular on answering two research questions: **(1)** when does such extensions of a partial AAM exist? and **(2)** how can such an extension be computed efficiently?

In the following Sect.  we provide the necessary theoretical framework, establishing the equivalence of AAMs and ITS graphs and introducing the *remainder graphs*. It is shown, moreover, that certain isomorphisms of these remainder graphs are a sufficient condition for the construction of equivalent of AAMs. We then consider reaction centers and their associated partial AAMs. The main result of Sect.  establishes that *good* partial AAMs are characterized by the existence of a unique *stable extension*, i.e., extensions preserving the reaction mechanism, that are in turn isomorphisms between remainder graphs, providing, therefore, the basis for our algorithms for the completion of good partial AAMs in Sect. . Additional complications arise if the AAM does not fully cover the reaction center. This is the case, in particular, of hydrogen atoms that are not explicitly represented in the reaction data, even when these play a key role for the reaction mechanism. In Sect. 27 we consider extensions of our framework to this case and establish further characterization results under the assumption that there are no isomorphisms that “mix” atoms covered by a given partial AAM with those appearing in extended graph representations. Empirical performance results are collected and discussed in Sect. 10.

## Reactions, AAMs and ITS graphs

### Basic Notation

**Molecules** are modeled as connected, labeled simple graphs with vertices representing atoms and edges corresponding to bonds. We write *V*(*G*) and *E*(*G*) for the vertex and edge set of a graph *G*. Atom types and bond types are specified by labeling functions $a_G:V(G) \rightarrow L_V$ and $b_G: E(G) \rightarrow L_E$, respectively, for non-empty and disjoint label sets $L_V$ and $L_E$. We reserve a special symbol $\otimes \notin L_V \cup L_E$ for our constructions (as in Definition 4 below). Charges, unpaired electrons (radicals) that are associated with an atom, are annotated in the vertex labels. Thus $L_V$ may contain, for example, H, $\text {H}^{+}$, and $\text {H}^{-}$ as different labels. Alternatively, this information, together with non-bonding electron pairs, can be associated with loops, see [21]. For ease of presentation we will not consider charges or unpaired electrons explicitly. The theory developed here does include these features, however. For standard terminology on Graph Theory, e.g. adjacency, connectedness and multiple-edges we refer to [22]. Two labeled graphs $G = (V(G), E(G), a_G, b_G)$ and $H = (V(H), E(H), a_H, b_H)$ are *isomorphic* if there is a bijection $\varphi : V(G) \rightarrow V(H)$ that preserves adjacency as well as vertex and edge labels. A map $\varphi :V(G)\rightarrow V(H)$ thus is an isomorphism if: (*i*) it is bijective, (*ii*) $\varphi (x)\varphi (y)\in E(H)$ if and only if $xy\in E(G)$, (*iii*) $a_H(\varphi (x)) = a_G(x)$ for all $x\in V(G)$ and (*iv*) $b_H(\varphi (x)\varphi (y)) = b_G(xy)$ for all $xy\in E(G)$. We write $\operatorname {Iso}(G,H)$ for the set of all isomorphisms from *G* to *H*, and $G \simeq H$ whenever *G* and *H* are isomorphic. Isomorphisms of *G* to itself are called automorphisms and form an algebraic group denoted $\operatorname {Aut}(G)$ when endowed the usual composition of functions. We express the composition of functions $\alpha : X \rightarrow Y$ and $\beta : Y \rightarrow Z$ as $\beta \alpha (x) = \beta \circ \alpha (x) :=\beta (\alpha (x))$.

**Reactions**, denoted as $G \longrightarrow H$, are pairs of graphs whose connected components represent the reactant and product molecules, respectively. Note that both *G* and *H* may contain multiple copies of isomorphic connected components depending on the stoichiometry of the the reaction. A reaction is *balanced* if there is an AAM for it, that is, there exists a bijection $\alpha :V(G)\rightarrow V(H)$ preserving atom-labels, i.e., $a_H(\alpha (x))=a_G(x)$ for all $x\in V(G)$. If charges and unpaired electrons are annotated as part of the vertex labels, we may allow $a_H(\alpha (x))\ne a_G(x)$. Of course the chemical element of each atom still must be preserved. From here on we ignore charges, etc., in our presentation. Thus we say $G \longrightarrow H$ is balanced if and only if for each atom type $c\in L_V$ we have $|a_{G}^{-1}(c)| = |a_{H}^{-1}(c)|$, i.e., reactants and products contain the same number of atoms of each type. Consider an AAM $\alpha$ for a balanced reaction $G \longrightarrow H$ and $xy \in E(G)$. We say that *xy* is a *reaction edge* of *G* induced by $\alpha$, if either $\alpha (x)\alpha (y) \notin E(H)$, or if $b_H(\alpha (x)\alpha (y)) \ne b_G(xy)$ whenever $\alpha (x)\alpha (y) \in E(H)$. Equally we say that $uv \in E(H)$ is a reaction edge of *H* induced by $\alpha$ if $\alpha ^{-1}(u)\alpha ^{-1}(v) \notin E(G)$, or $b_G(\alpha ^{-1}(u)\alpha ^{-1}(v)) \ne b_H(uv)$ provided $\alpha ^{-1}(u)\alpha ^{-1}(v) \in E(G)$. A vertex $x\in V(G)$ is a *reacting* vertex (for $\alpha$) if *x* is incident with a reaction edge in *G* or $\alpha (x)$ is incident with a reaction edge in *H*. Analogously, $x\in V(H)$ is a reacting vertex (for $\alpha$) if *x* is incident with a reaction edge in *H* or $\alpha ^{-1}(x)$ is incident with a reaction edge in *G*. In particular, therefore, both ends of a reaction edge are reacting vertices.

#### Definition 2

Let $\alpha$ be an AAM for the balanced reaction $G \longrightarrow H$. The *remainder graph* of *G* under $\alpha$, denoted $\hat{G}_{\alpha }$, is the subgraph of *G* obtained by removing all reaction edges of *G*, and preserving all vertex labels and all remaining edge labels. The remainder graph $\hat{H}_{\alpha }$ of *H* under $\alpha$ is defined analogously.

All non-reaction edges are by construction preserved between *G* and *H*. That is, if $xy\in E(G)$ is not a reaction edge then $\alpha (x)\alpha (y)\in E(H)$ and, *vice versa*, if $uv\in E(H)$ is not a reaction edge in *H*, then $\alpha (x)\alpha (y)\in E(H)$. As an immediate consequence we obtain,

#### Lemma 1

[16, Lemma 1] Every AAM $\alpha$ for a balanced reaction $G \longrightarrow H$ is an isomorphism from the remainder graph $\hat{G}_{\alpha }$ to the remainder graph $\hat{H}_{\alpha }$.

### Equivalent AAMs and Isomorphic ITS graphs

The problem of determining whether two AAMs $\alpha$ and $\beta$ for the same reaction are actually “the same” arises, for example, when comparing the results of different reaction mapping tools, because each tools uses its own graph representation. As a consequence, for a formal treatment of this problem it becomes necessary to compare $\alpha :V(G)\rightarrow V(H)$ and $\beta :V(G')\rightarrow V(H')$ given that *G* and $G'$, as well as *H* and $H'$, respectively, are isomorphic,

#### Definition 3

[4] Let $\alpha$ and $\beta$ be AAMs for, respectively, the balanced reactions $G \longrightarrow H$ and $G' \longrightarrow H'$ with $G' \simeq G$ and $H' \simeq H$. We say that $\alpha$ and $\beta$ are *equivalent*, denoted as $\alpha \equiv \beta$, if there exist isomorphisms $\varphi \in \operatorname {Iso}(G, G')$ and $\psi \in \operatorname {Iso}(H, H')$ such that $\psi \alpha = \beta \varphi$.

There is a close connection between the AAM $\alpha$ and isomorphisms on the remainder graphs that plays a central role, in particular, for Theorem 5, which is the main result supporting the methods of our contribution,

#### Proposition 1

Let $\alpha$ be an AAM for the balanced reaction $G \longrightarrow H$ and let $\beta$ be an isomorphism from the remainder graph $\hat{G}_{\alpha }$ to the remainder graph $\hat{H}_{\alpha }$. If $\alpha (x)=\beta (x)$ holds for all reacting vertices *x* of $\alpha$, then $\alpha$ and $\beta$ are equivalent AAMs for $G \longrightarrow H$.

#### Proof

To prove the equivalence of $\alpha$ and $\beta$ it suffices to show that $\beta ^{-1}\alpha \in \operatorname {Aut}(G)$. To see this note first that by Lemma 1 we have $\alpha \in \operatorname {Iso}(\hat{G}_{\alpha }, \hat{H}_{\alpha })$. Thus by inverses and composition of isomorphisms it follows $\beta ^{-1}\alpha \in \operatorname {Aut}(\hat{G}_{\alpha })$ and therefore, by definition, we get $a_G(x) = a_{\hat{G}_{\alpha }}(x) = a_{\hat{G}_{\alpha }}(\beta ^{-1}\alpha (x)) = a_G(\beta ^{-1}\alpha (x))$ for all $x \in V(G)$. Consider now $xy \in E(G)$. Then either $xy \in E(\hat{G}_{\alpha })$ or *xy* is a reaction edge of *G*. In the first case, from $\beta ^{-1}\alpha \in \operatorname {Aut}(\hat{G}_{\alpha })$ follows that **(A1)** $xy \in E(\hat{G}_{\alpha })$ if and only if $\beta ^{-1}\alpha (x)\beta ^{-1}\alpha (y) \in E(\hat{G}_{\alpha })$, and again $b_G(xy) = b_{\hat{G}_{\alpha }}(xy) = b_{\hat{G}_{\alpha }}(\beta ^{-1}\alpha (x)\beta ^{-1}\alpha (y)) = b_G(\beta ^{-1}\alpha (x)\beta ^{-1}\alpha (y))$. Suppose, on the other hand, that $xy \in E(G)$ is a reaction edge, i.e., $xy \in (E(G) {\setminus } E(\hat{G}_{\alpha }))$. Then, both *x* and *y* are reacting vertices, and by hypothesis $\alpha (x) = \beta (x)$ and $\alpha (y) = \beta (y)$, or equivalently $\beta ^{-1}\alpha (x) = x$ and $\beta ^{-1}\alpha (y) = y$. Thus formally we also have **(A2)** $xy \in (E(G) \setminus E(\hat{G}_{\alpha }))$ if and only if $\beta ^{-1}\alpha (x)\beta ^{-1}\alpha (y) \in (E(G) {\setminus } E(\hat{G}_{\alpha }))$ and similarly $b_G(xy) = b_G(\beta ^{-1}\alpha (x)\beta ^{-1}\alpha (y))$. In this way, from **(A1)** and **(A2)** we get $xy \in E(G)$ if and only if $\beta ^{-1}\alpha (x)\beta ^{-1}\alpha (y) \in E(G)$, i.e., $\beta ^{-1}\alpha$ preserves adjacency in *G*, and since it also preserves vertex and edge labels we have $\beta ^{-1}\alpha \in \operatorname {Aut}(G)$. Therefore, by setting $\varphi :=\beta ^{-1}\alpha \in \operatorname {Aut}(G)$ and $\psi :=i_H \in \operatorname {Aut}(H)$ for the identity automorphism $i_H: V(H) \rightarrow V(H)$, we get $\psi \alpha = i_H \alpha = (\beta \beta ^{-1})\alpha = \beta (\beta ^{-1}\alpha ) = \beta \varphi$ and thus $\alpha \equiv \beta$, proving the proposition. $\square$

The information in a balanced reaction $G\longrightarrow H$ and its AAM $\alpha$ can be compiled into the Imaginary Transition State (ITS) graph of the reaction by identifying atoms that correspond to each other via $\alpha$. The ITS graph thus contains the edges of both the reactants-graph *G* and the products-graph *H*. Both vertices and edges are associated with label pairs that derive from the labels in *G* and *H*. Formally this is,

#### Definition 4

Let $G \longrightarrow H$ be a balanced reaction with AAM $\alpha : V(G)\rightarrow V(H)$. An ITS graph *T* of $(G,H,\alpha )$ is a graph with vertex set *V*(*T*), edge set *E*(*T*), vertex-labeling function $a_T: V(T) \rightarrow L_V \times L_V$ and edge-labeling function $b_T: E(T) \rightarrow (L_E \cup \{\otimes \}) \times (L_E \cup \{\otimes \})$, obtained from *G* by means of a bijection $\tau : V(T)\rightarrow V(G)$ such that

(i) $x,y \in V(T)$ we have $xy \in E(T)$ if and only if $\tau (x)\tau (y) \in E(G)$ or $\alpha (\tau (x))\alpha (\tau (y))\in E(H)$;
(ii) each vertex $x \in V(T)$ is labeled by the ordered pair $a_T(x) = (a_G(\tau (x)),a_H(\alpha (\tau (x))))$,
(iii) each edge $xy \in E(T)$ is labeled by the ordered pair $b_T(xy)$ determined as follows:

$$\begin{aligned} b_T(xy)= {\left\{ \begin{array}{ll} (b_G(\tau (x)\tau (y)), b_H(\alpha (\tau (x))\alpha (\tau (y)))) &  \textit{ if } \tau (x)\tau (y)\in E(G) \textit{ and } \alpha (\tau (x))\alpha (\tau (y)) \in E(H) \\ (b_G(\tau (x)\tau (y)), \otimes ) &  \textit{ if } \tau (x)\tau (y)\in E(G) \textit{ and } \alpha (\tau (x))\alpha (\tau (y)) \notin E(H) \\ (\otimes , b_H(\alpha (\tau (x))\alpha (\tau (y)))) &  \textit{ if } \tau (x)\tau (y) \notin E(G) \textit{ and } \alpha (\tau (x))\alpha (\tau (y)) \in E(H) \\ \end{array}\right. } \end{aligned}$$

**Fig. 2:**
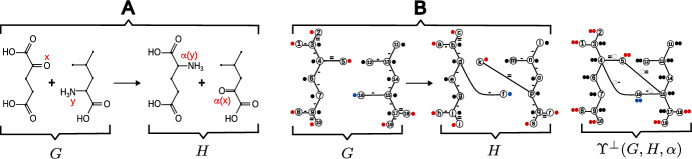
In **a** the reaction $G \longrightarrow H$ from Fig. 1 is shown with usual chemical notation. In **b** the same reaction is given another representation resembling our formalism. Here, vertices are labeled by element types: “O” , “N”  and “C” $\bullet$ (hydrogen is omitted), while edges are labeled with “=” and “-” for double and single bonds, respectively. The canonical ITS graph $\Upsilon ^{\perp }(G, H, \alpha )$ is shown on the right

Figure 2 below showcases the construction of an ITS graph. We will use, moreover, the notation $a_T(x)=(a_T^1(x),a_T^2(y))$ and $b_T(xy)=(b_T^1(xy),b_T^2(xy))$ to address the two components of the vertex and edge labels of an arbitrary ITS graphs.

For every balanced reaction with AAM $\alpha$ there exists an ITS graph. The construction is not unique, however, because of the arbitrary bijection $\tau$ between the vertices of *G* and the vertices of *T*. We note that the vertices of the ITS graph also bijectively map to *H* since for every $y \in V(H)$ there is a unique $v = \alpha ^{-1}(y) \in V(G)$ and $x = \tau ^{-1}(v) \in V(T)$, from where $y = \alpha (v) = \alpha (\tau (x)) = \tau '(x) \in V(H)$. Now we confirm, nonetheless, our intuition that ITS graphs are unique up to isomorphism,

#### Lemma 2

Let $\Upsilon ^{\circledast }(G, H, \alpha )$ be the (non-empty) collection of all ITS graphs built for a balanced reaction $G \longrightarrow H$ with AAM $\alpha$ and consider a graph $T \in \Upsilon ^{\circledast }(G, H, \alpha )$. Then $T' \in \Upsilon ^{\circledast }(G, H, \alpha )$ if and only if $T' \simeq T$.

#### Proof

Suppose first that $T' \in \Upsilon ^{\circledast }(G, H, \alpha )$. Let $\tau$ and $\tau '$ be the bijections required by Definition 4 for *T* and $T'$, respectively.

Consider two arbitrary vertices *u* and *v* in *V*(*G*) and their preimages $\tau ^{-1}(u)$ and $\tau ^{-1}(v)$ in *V*(*T*). Condition (*i*) in the definition can be restated for *u* and *v* as $\tau ^{-1}(u)\tau ^{-1}(v) \in E(T)$, if and only if, $uv \in E(G)$ or $\alpha (u)\alpha (v)\in E(H)$. At the same time, for two vertices $x, y \in V(T')$ it holds $xy \in E(T')$, if and only if, $\tau '(x)\tau '(y) \in E(G)$ or $\alpha (\tau '(x))\alpha (\tau '(y))\in E(H)$. From these statements it follows that $xy \in E(T')$ if and only if $\tau ^{-1}(\tau '(x))\tau ^{-1}(\tau '(y)) \in E(T)$, i.e., the bijection $\tau ^{-1}\tau ': V(T') \rightarrow V(T)$ preserves adjacency between *T* and $T'$. We see, moreover, that $a_{T}(\tau ^{-1}\tau '(x)) = (a_G(\tau (\tau ^{-1}\tau '(x))), a_H(\alpha (\tau (\tau ^{-1}\tau '(x))))) = (a_G(\tau '(x)), a_H(\alpha (\tau '(x)))) = a_{T'}(x)$, thus the map $\tau ^{-1}\tau '$ preserves vertex labels. Similarly, and without loss of generality, we have

$$b_T(\tau ^{-1}\tau '(x)\tau ^{-1}\tau '(y)) = (b_G(\tau (\tau ^{-1}\tau '(x))\tau (\tau ^{-1}\tau '(y))), b_H(\alpha (\tau (\tau ^{-1}\tau '(x))))\alpha (\tau (\tau ^{-1}\tau '(x)))) = \ldots $$

$\ldots = (b_G(\tau '(x)\tau '(y)), b_H(\alpha (\tau '(x))\alpha (\tau '(x)))) = b_{T'}(xy)$, that is, $\tau ^{-1}\tau '$ also preserves edge labels and is, therefore, an isomorphism from $T'$ to *T*, i.e., $T' \simeq T$ proving the forward direction.

Suppose, on the other hand, that there exist an isomorphism $\varphi : V(T') \rightarrow V(T)$ from $T'$ to *T*. Given that $\varphi$ preserves adjacency, vertex-labels and edge-labels, it follows that: (i) $xy \in E(T')$, if and only if, $\varphi (x)\varphi (y) \in E(T')$, and equivalently, $\tau (\varphi (x))\tau (\varphi (y)) \in E(G)$ or $\alpha (\tau (\varphi (x)))\alpha (\tau (\varphi (y)))\in E(H)$, also (ii) $a_{T'}(x) = a_T(\varphi (x)) = (a_G(\tau (\varphi (x))), a_H(\alpha (\tau (\varphi (y)))))$, and lastly (iii) $b_{T'}(xy) = a_T(\varphi (x)\varphi (y)) = (b_G(\tau (\varphi (x))\tau (\varphi (y))), b_H(\alpha (\tau (\varphi (x)))\alpha (\tau (\varphi (y)))))$. Thus $\tau \varphi : V(T') \rightarrow V(G)$ satisfies all the conditions required by Definition 4 for $T'$ and therefore $T' \in \Upsilon ^{\circledast }(G, H, \alpha )$, which proves the converse statement. $\square$

Thus it suffices to consider an arbitrary representative ITS from $\Upsilon ^{\circledast }(G, H, \alpha )$, which we will denote by $\Upsilon (G,H,\alpha )$. In earlier work, moreover, the ITS graph is defined by setting $V(T)=V(G)$ and using the identity map on *G* for $\tau$, see e.g. [4]. We will denote this (unique) particular representative, that we call *canonical ITS graph*, by $\Upsilon ^{\perp } :=\Upsilon ^{\perp }(G, H, \alpha )$, see Fig. 2. The uniqueness of $\Upsilon ^{\perp }$ follows immediately from Definition 4. To see this suppose that there exist two such ITS graphs *T* and $T'$. Substituting $\tau$ with the identity map on *G* we get from (*i*) that $xy \in E(T)$ if and only if $xy \in E(T')$ for all $x, y \in V(T) = V(G)$, and from (*ii*) and (*iii*) it follows, respectively, $a_T = a_{T'}$ and $b_T = b_{T'}$.

In [4], we proved the statement of the following Corollary for $\Upsilon ^{\perp }$. With Lemma 2, on the other hand, we can now restate this result for arbitrary representatives,

#### Corollary 1

[4, Cor.1] Let $G\longrightarrow H$ and $G'\longrightarrow H'$ be balanced reactions with AAMs $\alpha :V(G)\rightarrow V(H)$ and $\beta :V(G')\rightarrow V(H')$ and assume $G'\simeq G$ and $H'\simeq H$. Then $\alpha \equiv \beta$ if and only if $\Upsilon (G,H,\alpha ) \simeq \Upsilon (G',H',\beta )$ holds for any pair of ITS graphs for the two reactions.

Corollary 1 shows that each equivalence class of AAMs for a reaction $G \longrightarrow H$ produces a unique equivalence class of isomorphic ITS graph representations, provided that the AAMs being compared are defined over reactions $G' \longrightarrow H'$ with isomorphic reactants $G' \simeq G$ and products $H' \simeq H$. It is natural to ask whether there exist isomorphic ITS graphs $\Upsilon (G,H,\alpha )$ and $\Upsilon (G',H',\beta )$ for reactions with non-isomorphic graphs $G' \not \simeq G$ or $H' \not \simeq H$. The following result shows that this is indeed not possible,

#### Proposition 2

Let $\alpha$ and $\beta$ be AAMs for, respectively, two balanced reactions $G \longrightarrow H$ and $G' \longrightarrow H'$, and let $\Upsilon (G, H, \alpha )$ and $\Upsilon (G', H', \beta )$ be their corresponding ITS representations. Then $\Upsilon (G,H,\alpha ) \simeq \Upsilon (G',H',\beta )$ if and only if $G' \simeq G$, $H' \simeq H$, and $\alpha \equiv \beta$.

#### Proof

Note that by Lemma 2 and by the transitivity of the isomorphism relation $\simeq$, from $\Upsilon (G, H, \alpha ) \simeq \Upsilon (G', H', \beta )$, it follows that $\Upsilon ^{\perp }(G, H, \alpha ) \simeq \Upsilon ^{\perp }(G', H', \beta )$ holds for the canonical ITS representations $\Upsilon ^{\perp }_{\alpha } :=\Upsilon ^{\perp }(G, H, \alpha )$ of $(G, H, \alpha )$ and $\Upsilon ^{\perp }_{\beta } :=\Upsilon ^{\perp }(G', H', \beta )$ of $(G', H', \beta )$, having $V(\Upsilon ^{\perp }_{\alpha }) = V(G)$ and $V(\Upsilon ^{\perp }_{\beta }) = V(G')$, and for which the identity maps $i_G$ over *V*(*G*) and $i_{G'}$ over $V(G')$ satisfy Definition 4, respectively. Consider an isomorphism $\varphi \in \operatorname {Iso}(\Upsilon ^{\perp }_{\alpha }, \Upsilon ^{\perp }_{\beta })$, and note that this is also a bijection $\varphi : V(G) \rightarrow V(G')$. Thus when applying condition (*i*) in Definition 4 and given that $\varphi$ preserves adjacency between $\Upsilon ^{\perp }_{\alpha }$ and $\Upsilon ^{\perp }_{\beta }$ it follows that, $xy \in E(G)$ or $\alpha (x)\alpha (y) \in E(H)$, if and only if, $\varphi (x)\varphi (y) \in E(G')$ or $\beta (\varphi (x))\beta (\varphi (y)) \in E(H')$. This suggests that the bijections $\varphi$ and $\beta \varphi \alpha ^{-1}: V(H) \rightarrow V(H')$ are the required isomorphisms $\varphi \in \operatorname {Iso}(G, G')$ and $\beta \varphi \alpha ^{-1} \in \operatorname {Iso}(H, H')$. To actually prove this, note first that under $\varphi$ all labels are preserved component-wise, i.e., for any vertices $x, y \in V(G)$ we have, **(P1)** $(a_{\Upsilon ^{\perp }_{\alpha }}^1(x), a_{\Upsilon ^{\perp }_{\alpha }}^2(x)) = a_{\Upsilon ^{\perp }_{\alpha }}(x) = a_{\Upsilon ^{\perp }_{\beta }}(\varphi (x)) = (a_{\Upsilon ^{\perp }_{\beta }}^1(\varphi (x)), a_{\Upsilon ^{\perp }_{\beta }}^2(\varphi (x)))$ and **(P2)** $(b_{\Upsilon ^{\perp }_{\alpha }}^1(xy), b_{\Upsilon ^{\perp }_{\alpha }}^2(xy)) = b_{\Upsilon ^{\perp }_{\alpha }}(xy) = b_{\Upsilon ^{\perp }_{\beta }}(\varphi (x)\varphi (y)) = (b_{\Upsilon ^{\perp }_{\beta }}^1(\varphi (x)\varphi (y)), b_{\Upsilon ^{\perp }_{\beta }}^2(\varphi (x)\varphi (y)))$. For *G* and $G'$, **(P1)** implies $a_G(x) = a_{\Upsilon ^{\perp }_{\alpha }}^1(x) = a_{\Upsilon ^{\perp }_{\beta }}^1(\varphi (x)) = a_{G'}(\varphi (x))$, while from **(P2)** we get $b_{\Upsilon ^{\perp }_{\alpha }}^1(xy) = b_{\Upsilon ^{\perp }_{\beta }}^1(\varphi (x)\varphi (y)) \in L_E \cup \{\otimes \}$, which by condition (*iii*) in the definition can only happen if $\varphi$ preserves adjacency, i.e., $xy \in E(G)$ if and only if $\varphi (x)\varphi (y) \in E(G')$, also yielding $b_G(xy) = b_{G'}(\varphi (x)\varphi (y))$ when the edges are present in these graphs. Thus $\varphi : V(G) \rightarrow V(G')$ preserves adjacency, vertex labels and edge labels, and therefore $\varphi \in \operatorname {Iso}(G, G')$. Similarly for *H* and $H'$, (P1) implies $a_H(\alpha (x)) = a_{\Upsilon ^{\perp }_{\alpha }}^2(\alpha (x)) = a_{\Upsilon ^{\perp }_{\beta }}^2(\beta (\varphi (x))) = a_{H'}(\beta (\varphi (x)))$, which we can rewrite as $a_H(v) = a_{H'}(\beta (\varphi (\alpha ^{-1}(v))))$ for $v :=\alpha (x) \in V(H)$, thus $\beta \varphi \alpha ^{-1}$ preserves vertex labels. Since from (P2) it holds $b_{\Upsilon ^{\perp }_{\alpha }}^2(\alpha (x)\alpha (y)) = b_{\Upsilon ^{\perp }_{\beta }}^2(\beta (\varphi (x))\beta (\varphi (y))) \in L_E \cup \{\otimes \}$, we have $b_H(\alpha (x)\alpha (y)) = b_{H'}(\beta (\varphi (x))\beta (\varphi (y)))$, and equivalently $b_H(uv) = b_{H'}(\beta (\varphi (\alpha ^{-1}(u)))\beta (\varphi (\alpha ^{-1}(v))))$ for $v :=\alpha (x)$ and $u :=\alpha (y)$ in *V*(*H*), whenever the respective edges are present, while in general, from this together with condition (*iii*) in Definition 4, we get again $\alpha (x)\alpha (y) \in E(H)$ if and only if $\beta (\varphi (x))\beta (\varphi (y)) \in E(H')$, or equivalently, $uv \in E(H)$ if and only if $\beta (\varphi (\alpha ^{-1}(u)))\beta (\varphi (\alpha ^{-1}(v))) \in E(H')$. This shows that $\beta \varphi \alpha ^{-1}$ preserves adjacency, and vertex and edge labels, and thus $\beta \varphi \alpha ^{-1} \in \operatorname {Iso}(H, H')$. Lastly, since $G' \simeq G$ and $H' \simeq H$ hold, the hypothesis $\Upsilon (G, H, \alpha ) \simeq \Upsilon (G', H', \beta )$, together with Corollary 1, now implies also that $\alpha \equiv \beta$. The converse statement follows from Corollary 1. $\square$

### Reaction Centers

In Sect.  we defined reaction edges and reacting vertices for a reaction $G \longrightarrow H$ with a given AAM $\alpha$. Since *G*, *H*, and $\alpha$ are uniquely defined up to graph isomorphisms and equivalence of AAMs by any representative ITS graph $\Upsilon (G,H,\alpha )$, these definitions carry over ITS graphs, i.e., they also have (a version of) reaction edges and reacting vertices. ITS graphs contain, moreover, an isomorphic copy of the remainder graphs $\hat{G}_{\alpha }$ and $\hat{H}_{\alpha }$,

#### Lemma 3

Let $\Upsilon :=\Upsilon (G, H, \alpha )$ be an ITS representation of the balanced reaction $G \longrightarrow H$ with AAM $\alpha$ and let $\eta : V(G)\rightarrow V(\Upsilon )$ and $\eta ':=\eta \circ \alpha ^{-1}: V(H) \rightarrow V(\Upsilon )$ be the corresponding bijections that embed *G* and *H* into $\Upsilon$, i.e., where $\eta :=\tau ^{-1}$ for $\tau : V(\Upsilon ) \rightarrow V(G)$ as required by Definition 4. Then the following hold,

(i) $xy \in E(G)$ is a reaction edge in *G* if and only if $b_{\Upsilon }^1(\eta (x)\eta (y))\ne b_{\Upsilon }^2(\eta (x)\eta (y))$, and $x'y' \in E(H)$ is a reaction edge in *H* if and only if $b_{\Upsilon }^1(\eta '(x')\eta '(y')) \ne b_{\Upsilon }^2(\eta '(x')\eta '(y'))$.
(ii) $xy \in E(\hat{G}_{\alpha })$, and thus also $\alpha (x)\alpha (y) \in E(\hat{H}_{\alpha })$, if and only if, $\eta (x)\eta (y) \in E(\Upsilon )$ and $b_{\Upsilon }^1(\eta (x)\eta (y)) = b_{\Upsilon }^2(\eta '(\alpha (x))\eta '(\alpha (y)))$.

#### Proof

Set $\eta :=\tau ^{-1}$ for $\tau$ as in Definition 4. This implies that $\eta (x)\eta (y)\in E(\Upsilon )$ if and only if $xy \in E(G)$ or $\alpha (x)\alpha (y) \in E(H)$. The definition of the edge labels and the bijection $\eta ': V(H) \rightarrow V(\Upsilon )$ now yield $b_{\Upsilon }(\eta (x)\eta (y)) = b_{\Upsilon }(\eta '(\alpha (x))\eta '(\alpha (y))) = (b_G(xy), b_H(\alpha (x)\alpha (y)))$. Recall that *xy* is a reaction edge in *G* if either, $\alpha (x)\alpha (y) \in E(H)$ in which case $b_G(xy) \ne b_H(\alpha (x)\alpha (y))$, or $\alpha (x)\alpha (y) \notin E(H)$ and thus $b_{\Upsilon }(\eta (x)\eta (y)) = (b_G(xy), \varnothing ,)$. In either case Definition 4 yields $b_{\Upsilon }^1(\eta (x)\eta (y)) \ne b_{\Upsilon }^2(\eta (x)\eta (y))$. The same argument can be made for edges $\alpha (x)\alpha (y)\in E(H)$. The second statement now follows directly from Definition 2 since the remainder graph $\hat{G}_{\alpha }$ contains exactly the edges with $b_G(xy) = b_H(\alpha (x)\alpha (y))$ and hence $b_{\Upsilon }^1(\eta (x)\eta (y)) = b_{\Upsilon }^2(\eta '(\alpha (x))\eta '(\alpha (y)))$. $\square$

We then refer to the edges $uv\in E(\Upsilon (G,H,\alpha ))$ with unequal labels, i.e., with $b_{\Upsilon }^1(uv) \ne b_{\Upsilon }^2(uv)$, simply as reaction edges. Reacting vertices of $(G,H,\alpha )$ are thus represented in the ITS graph as the vertices incident with edges that have unequal labels. The reaction center of a reaction $G \longrightarrow H$ comprises all the bonds modified by the electron exchange occurring during the reaction. This notion appeared already in [23–25] and was used in [26] to classify reaction types. Its formal properties were studied in more detail in [16] and [21].

#### Definition 5

Let $\Upsilon (G,H,\alpha )$ be an ITS representation of the balanced reaction $G \longrightarrow H$ with AAM $\alpha$. The *reaction center* is the subgraph $\Gamma (G,H,\alpha )$ of $\Upsilon (G,H,\alpha )$ comprising all the reaction edges and reacting vertices.

It follows immediately from Prop. 2 that $\Gamma (G, H, \alpha ) \simeq \Gamma (G', H', \beta )$ whenever $G \simeq G'$, $H \simeq H'$, and $\alpha \equiv \beta$. The converse statement, however, is not true in general. We show examples about this in [16], of graphs with isomorphic reaction centers, and (*i*) with $G \simeq G'$ but $H \not \simeq H'$ (Fig. 8 in [16]), and (*ii*) with $G \simeq G'$ and $H \simeq H'$ but $\alpha \not \equiv \beta$ (Fig. 10 in [16]). We will write, moreover, $\Gamma ^{\perp } :=\Gamma ^{\perp }(G, H, \alpha )$ for the reaction center of the canonical representation $\Upsilon ^{\perp }(G, H, \alpha )$ of the triple $(G, H, \alpha )$. It is worth mentioning that in [16] we considered the connectedness of these graphs. Though in general the connectedness of an ITS graph does not guarantee the connectedness of its reaction center or *vice versa* (see Figure 2 of [16]), we will, in practice, restrict ourselves to single-step reactions, which have connected reaction centers, i.e., a disconnected reaction center models two independent reactions. The following result, therefore, will also be of relevance for our algorithmic approach:

#### Lemma 4

[16], Lemma 2] Let $\alpha$ be an AAM for a balanced reaction $G \longrightarrow H$. If $\Upsilon (G, H, \alpha )$ is a connected graph, then every connected component of *G* and *H* contains at least one reacting vertex.

### Partial AAMs and their Partial ITS graphs

Theoretically speaking, both matches $\mu$ and $\nu$ of a reaction template, as in Definition 1, generally provide only a *partial AAM*. This is also the case, in practice, of computational mapping tools such as LocalMapper [13], which can focus on only determining a most plausible reaction center and necessary adjacent context.

#### Definition 6

Let $G \longrightarrow H$ be a reaction. A *partial AAM* is a bijection $\pi : U \rightarrow W$, for two subsets $U \subseteq V(G)$ and $W \subseteq V(H)$, which preserves vertex labels, i.e., such that $a_H(\pi (x)) = a_G(x)$ for all $x\in U$. Moreover, if $U \subsetneq V(G)$ and $W \subsetneq V(H)$, then we say that $\pi$ is a *proper partial AAM* for the reaction $G \longrightarrow H$.

Given a reaction $G \longrightarrow H$ and a partial AAM $\pi :U\rightarrow W$, it is immediate that the ITS graph $\Upsilon (G[U],H[W],\pi )$, together, in particular, with the canonical graphs $\Upsilon ^{\perp }(G[U],H[W],\pi )$ and $\Gamma ^{\perp }(G[U],H[W],\pi )$, are all well-defined, see Fig. 4 for an example.

#### Definition 7

Let $G \longrightarrow H$ be a balanced reaction and let $\pi :U\rightarrow W$ with $U \subseteq V(G)$ and $W \subseteq V(H)$ be a partial AAM. Then an AAM $\alpha : V(G) \rightarrow V(H)$ is said to be an *extension* of $\pi$, or to *extend* $\pi$, if $\alpha (x)=\pi (x)$ for all $x \in U$.

As noted in [16, Obs. 3], every partial AAM $\pi$ for $G \longrightarrow H$ has an extension $\alpha$. Moreover, it follows directly from the definition that the partial ITS graph $\Upsilon (G[U],H[W],\pi )$ representing $\pi$ is isomorphic to the subgraph of the canonical ITS graph of $(G,H,\alpha )$ induced by *U*, i.e., $\Upsilon (G[U],H[W],\pi ) \simeq \Upsilon ^{\perp }(G,H,\alpha )[U]$. This isomorphism, furthermore, becomes the identity $\Upsilon ^{\perp }(G[U],H[W],\pi ) = \Upsilon ^{\perp }(G,H,\alpha )[U]$ for the respective canonical ITS graphs, while for the canonical reaction centers we get $\Gamma ^{\perp }(G[U],H[W],\pi ) \subseteq \Gamma ^{\perp }(G,H,\alpha )$. In general, therefore, $\Upsilon ^{\perp }(G[U],H[W],\pi )$ will not contain all reaction edges. For many application scenarios, in particular the ones mentioned in the introduction, we do expect this to be the case. It is of interest, therefore, to determine whether a partial AAM, and thus its corresponding partial ITS graphs, already contains all reaction edges.

#### Definition 8

A partial AAM $\pi : U \rightarrow W$ with $U \subseteq V(G)$ and $W \subseteq V(H)$ for a balanced reaction $G \longrightarrow H$ is said to be a *good* partial AAM, if there is an extension $\alpha :V(G)\rightarrow V(H)$ of $\pi$ such that $\Gamma ^{\perp }(G, H,\alpha ) = \Gamma ^{\perp }(G[U],H[W],\pi )$. If such extension does not exists, we say that $\pi$ is a *bad* partial AAM.

In other words, $\pi$ is a good partial map for a balanced reaction if and only if there is an extension $\alpha$ of $\pi$, such that the induced subgraph $\Upsilon ^{\perp }(G,H,\alpha )[U]$ of the canonical ITS graph, contains all reaction edges of $\Upsilon ^{\perp }(G,H,\alpha )$. In this case we call $\alpha$ a *stable extension* of $\pi$.

We have analyzed both good and bad partial AAMs in previous work [16]. In this section we focus, specifically, on the study of the extension of good partial AAMs into full AAMs and its relation to the remainder graphs.

Recall that $\hat{G}_{\alpha }$ and $\hat{H}_{\alpha }$ denote the remainder graphs obtained from *G* and *H* with respect to a full AAM $\alpha$ (see Definition 2). In order to better understand stable extensions we need to consider, additionally, the *remainder* graphs $\hat{G}_{\pi }$ and $\hat{H}_{\pi }$ induced by $\pi$, preserving vertex and edge labels of *G* and *H*, but obtained by removing from them, respectively, those reaction edges induced by $\pi : U \rightarrow W$ for *G*[*U*] and *H*[*W*], i.e., edges $xy \in E(G[U])$ such that $\pi (x)\pi (y) \notin E(H[W])$, or $\pi (x)\pi (y) \in E(H[W])$ but with $b_G(xy) \ne b_H(\pi (x)\pi (y))$, and edges $uv \in E(H[W])$ with $\pi ^{-1}(u)\pi ^{-1}(v) \notin E(G[U])$, or such that $\pi ^{-1}(u)\pi ^{-1}(v) \in E(G[U])$ but with $b_G(\pi ^{-1}(u)\pi ^{-1}(v)) \ne b_H(uv)$. Consider an arbitrary extension $\alpha$ of $\pi$. Clearly $V(\hat{G}_{\pi }) = V(G) = V(\hat{G}_{\alpha })$ and $V(\hat{H}_{\pi }) = V(H) = V(\hat{H}_{\alpha })$. Moreover, since $\alpha$ produces at least the same reaction edges as $\pi$, from these definitions we immediately get

#### Proposition 3

Let $\alpha$ be an arbitrary extension of a partial AAM $\pi$ for a balanced reaction $G \longrightarrow H$. Then, $E(\hat{G}_{\alpha }) \subseteq E(\hat{G}_{\pi })$ and $E(\hat{H}_{\alpha }) \subseteq E(\hat{H}_{\pi })$.

Based on this simple observation, the following two results strengthen the connection between the remainder graphs induced by full and partial AAMs:

#### Lemma 5

Let $G \longrightarrow H$ be a balanced reaction and $\pi : U\rightarrow W$ with $U\subseteq V(G)$ and $W\subseteq V(H)$ be a partial AAM. Consider an extension $\alpha$ of $\pi$. Then, $\pi$ is a good partial AAM with stable extension $\alpha$, if and only if, $\hat{G}_{\alpha } = \hat{G}_{\pi }$ and $\hat{H}_{\alpha } = \hat{H}_{\pi }$.

#### Proof

Suppose first that $\pi$ is a good partial AAM with stable extension $\alpha$. Thus, by definition we have $\Gamma ^{\perp }(G, H,\alpha ) = \Gamma ^{\perp }(G[U],H[W],\pi )$. Consider then a reaction edge *xy* of *G* induced by $\alpha$. By taking, in Lemma 3, the bijection $\eta$ (resp. $\tau$) to be the identity mapping on *G*, it follows that *xy* is an edge in $\Upsilon ^{\perp }: = \Upsilon ^{\perp }(G, H,\alpha )$ with $b_{\Upsilon ^{\perp }}^1(xy) \ne b_{\Upsilon ^{\perp }}^2(xy)$, and therefore $xy \in \Gamma ^{\perp }(G, H,\alpha )$.

Then *xy* is also a reaction edge of $\Upsilon ^{\perp }(G[U],H[W],\pi )$, and by another application of Lemma 3 we conclude that *xy* is also a reaction edge of *G*[*U*]. By contraposition, moreover, this implies that if $xy \in E(\hat{G}_{\pi })$, then $xy \in E(\hat{G}_{\alpha })$, proving the contention $E(\hat{G}_{\pi }) \subseteq E(\hat{G}_{\alpha })$, which together with Proposition 3, yields $E(\hat{G}_{\alpha }) = E(\hat{G}_{\pi })$ and thus $\hat{G}_{\alpha } = \hat{G}_{\pi }$. By similar arguments, for a reaction edge *uv* of *H* induced by $\alpha$ it follows that $\alpha ^{-1}(u)\alpha ^{-1}(v)$ is an edge in $\Upsilon ^{\perp }$ with $b_{\Upsilon ^{\perp }}^1(\alpha ^{-1}(u)\alpha ^{-1}(v)) \ne b_{\Upsilon ^{\perp }}^2(\alpha ^{-1}(u)\alpha ^{-1}(v))$. Then $\alpha ^{-1}(u)\alpha ^{-1}(v)$ is also an edge in $\Gamma ^{\perp }(G[U],H[W],\pi )$, implying that $\alpha ^{-1}(u), \alpha ^{-1}(v) \in U$. But $\alpha$ and $\pi$ coincide for all vertices *U*, and so $\alpha ^{-1}(u) = \pi ^{-1}(u)$ and $\alpha ^{-1}(v) = \pi ^{-1}(u)$, that is, $\pi ^{-1}(u)\pi ^{-1}(v)$ is an edge in $\Upsilon ^{\perp }(G[U],H[W],\pi )$ with $b_{\Upsilon ^{\perp }}^1(\pi ^{-1}(u)\pi ^{-1}(v)) \ne b_{\Upsilon ^{\perp }}^2(\pi ^{-1}(u)\pi ^{-1}(v))$ and thus, applying Lemma 3, we see that *uv* is also a reaction edge of *H* induced by $\pi$. By contraposition this also proves the inclusion $E(\hat{H}_{\pi }) \subseteq E(\hat{H}_{\alpha })$. Proposition 3 implies $\hat{H}_{\alpha } = \hat{H}_{\pi }$, proving the forward direction.

For the proof of the converse statement note that the hypotheses $\hat{G}_{\alpha } = \hat{G}_{\pi }$ and $\hat{H}_{\alpha } = \hat{H}_{\pi }$, imply that $\alpha$ and $\pi$ induce the same reaction edges for each *G* and *H*, that is, **(R1)** *xy* is a reaction edge of *G* w.r.t $\alpha$ if and only if *xy* is a reaction edge of *G* w.r.t $\pi$ and similarly **(R2)** *uv* is a reaction edge of *H* w.r.t $\alpha$ if and only if *uv* is a reaction edge of *H* w.r.t $\pi$.

This implies, by definition of the remainder graphs, that the vertices $x, y, \alpha ^{-1}(u), \alpha ^{-1}(v)$ are all in *U*, and thus $\alpha$ and $\pi$ coincide for each of the two ends of every reaction edge of *G* and/or *H*. Thus, denoting $\Upsilon ^{\perp }_{\alpha } :=\Upsilon ^{\perp }(G, H,\alpha )$ and $\Upsilon ^{\perp }_{\pi } :=\Upsilon ^{\perp }(G[U],H[W],\pi )$, through Lemma 3 condition **(R1)** implies **(R1’)** given $xy \in E(G)$, *xy* is a reaction edge of $\Upsilon ^{\perp }_{\alpha }$ if and only if *xy* is a reaction edge of $\Upsilon ^{\perp }_{\pi }$, i.e., *xy* is labeled by an ordered pair with different entries and, moreover, $b_{\Upsilon ^{\perp }_{\alpha }}(xy) = b_{\Upsilon ^{\perp }_{\pi }}(xy)$ since $x,y \in U$. Similarly, **(R2)** implies **(R2’)** given $uv \in E(H)$, $\alpha ^{-1}(u)\alpha ^{-1}(v)$ is a reaction edge of $\Upsilon ^{\perp }_{\alpha }$ if and only if $\alpha ^{-1}(u)\alpha ^{-1}(v)$ is a reaction edge of $\Upsilon ^{\perp }_{\pi }$, and again $b_{\Upsilon ^{\perp }_{\alpha }}(\alpha ^{-1}(u)\alpha ^{-1}(v)) = b_{\Upsilon ^{\perp }_{\pi }}(\alpha ^{-1}(u)\alpha ^{-1}(v))$ since $\alpha ^{-1}(u), \alpha ^{-1}(v) \in U$. But every reaction edge *xy* of $\Upsilon ^{\perp }_{\alpha }$, and $\Upsilon ^{\perp }_{\pi }$, is labeled by a pair $b_{\Upsilon ^{\perp }_{\bullet }}(xy) \in \{(a, \otimes ), (\otimes , b), (c, d)\}$ with $a, b, c, d \ne \otimes$, that is: *xy* is only a reaction edge of *G*, $\alpha (x)\alpha (y) = \pi (x)\pi (y)$ is only a reaction edge of *H*, or both edges are respective reaction edges of *G* and *H*. Thus **(R1’)** and **(R2’)** are exhaustive cases, and since the edge labels are the same in both $\Upsilon ^{\perp }_{\alpha }$ and $\Upsilon ^{\perp }_{\pi }$, we have $xy \in E(\Gamma ^{\perp }(G, H,\alpha ))$ if and only if $xy \in E(\Gamma ^{\perp }(G[U],H[W],\pi ))$ with $b_{\Gamma ^{\perp }_{\alpha }}(xy) = b_{\Gamma ^{\perp }_{\pi }}(xy)$. Thus $\Gamma ^{\perp }(G, H,\alpha ) = \Gamma ^{\perp }(G[U],H[W],\pi )$. Therefore $\pi$ is good and has $\alpha$ as stable extension. $\square$

#### Lemma 6

Let $G \longrightarrow H$ be a balanced reaction and $\pi : U\rightarrow W$ with $U\subseteq V(G)$ and $W\subseteq V(H)$ be a partial AAM. Consider an extension $\alpha$ of $\pi$. Then, $\alpha$ is an isomorphism from $\hat{G}_{\pi }$ to $\hat{H}_{\pi }$, if and only if, $\pi$ is a good partial AAM with stable extension $\alpha$.

#### Proof

Suppose first that $\alpha \in \operatorname {Iso}(\hat{G}_{\pi }, \hat{H}_{\pi })$. Consider any edge $xy \in E(\hat{G}_{\pi }) \subseteq E(G)$. Since $\alpha$ by assumption preserves adjacency, vertex labels and edge labels, we obtain $\alpha (x)\alpha (y) \in E(\hat{H}_{\pi }) \subseteq E(H)$. Since $\hat{G}_{\pi }$ and $\hat{H}_{\pi }$ take their edge labels from *G* and *H*, respectively, we also have $b_G(xy) = b_H(\alpha (x)\alpha (y))$, i.e., *xy* is not a reaction edge of *G* w.r.t $\alpha$ and thus $xy \in E(\hat{G}_{\alpha })$. Similarly, for $uv \in E(\hat{H}_{\pi }) \subseteq E(H)$, we get $\alpha ^{-1}(u)\alpha ^{-1}(v) \in E(\hat{G}_{\pi }) \subseteq E(G)$ and $b_G(\alpha ^{-1}(u)\alpha ^{-1}(v)) = b_H(uv)$, from where $uv \in E(\hat{H}_{\alpha })$. This shows $E(\hat{G}_{\pi }) \subseteq E(\hat{G}_{\alpha })$ and $E(\hat{H}_{\pi }) \subseteq E(\hat{H}_{\alpha })$. Then, Proposition 3 implies that $\hat{G}_{\pi } = \hat{G}_{\alpha }$ and $\hat{H}_{\pi } = \hat{H}_{\alpha }$, and from Lemma 5 it follows, therefore, that $\pi$ is good and $\alpha$ is a stable extension for $\pi$.

To prove the converse, suppose $\alpha$ is a stable extension for $\pi$. Then, by definition, $\alpha$ is also a full AAM for $G \longrightarrow H$, and by Lemma 1 we see that $\alpha$ is also an isomorphism from $\hat{G}_{\alpha }$ to $\hat{H}_{\alpha }$. But from Lemma 5 we should have $\hat{G}_{\pi } = \hat{G}_{\alpha }$ and $\hat{H}_{\pi } = \hat{H}_{\alpha }$, and therefore $\alpha \in \operatorname {Iso}(\hat{G}_{\pi }, \hat{H}_{\pi })$. $\square$

**Fig. 3:**
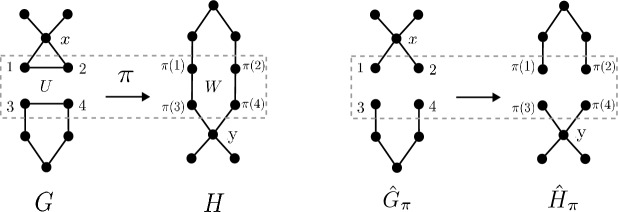
The remainder graphs $\hat{G}_{\pi }$ and $\hat{H}_{\pi }$ are isomorphic, and all isomorphisms between them must map the vertex $x \in V(\hat{G}_{\pi })$ to $y \in V(\hat{H}_{\pi })$, since these are the only vertices of degree 4 in them. Yet, the neighbors 1 and 2 of *x* in $\hat{G}_{\pi }$ are mapped by $\pi$ to a different component than that of *y* in $\hat{H}_{\pi }$. Therefore, no isomorphism between these graphs extends the partial AAM $\pi$, i.e., $\pi$ has no stable extensions

The hypothesis of Lemma 6 requires the isomorphism between the remainder graphs $\hat{G}_{\pi }$ and $\hat{H}_{\pi }$ to be an extension of the partial map $\pi$. The existence of an arbitrary isomorphism between $\hat{G}_{\pi }$ and $\hat{H}_{\pi }$ indeed is not sufficient for the existence of a stable extension for $\pi$, for a counterexample see Fig. 3.

With Lemmas 5 and 6, moreover, we recover the following results that we originally stated in [16, Prop. 1], now under the formalism of the canonical reaction center and canonical ITS graphs as in Definition 8:

#### Proposition 4

Let $\pi$ be a partial AAM for a balanced reaction $G \longrightarrow H$ and $\alpha$ an extension of $\pi$. The following statements are equivalent,

(i) $\pi$ is a good partial AAM and $\alpha$ is a stable extension of $\pi$,
(ii) $$\hat{G}_{\alpha } = \hat{G}_{\pi }$$ and $$\hat{H}_{\alpha } = \hat{H}_{\pi }$$,
(iii) $$\alpha \in \operatorname {Iso}(\hat{G}_{\pi }, \hat{H}_{\pi })$$.

### Uniqueness of Stable Extensions

Good partial AAMs can have multiple non-identical stable extensions. Prop. 4, on the other hand, implies that all of them are isomorphisms between the remainder graphs $\hat{G}_{\pi }$ and $\hat{H}_{\pi }$.

#### Theorem 5

Let $\pi$ be a good partial AAM for a balanced reaction $G \longrightarrow H$ and let $\alpha$ and $\beta$ be two stable extensions of $\pi$. Then $\alpha \equiv \beta$.

#### Proof

Suppose $\pi$ is the map $\pi : U \rightarrow W$ for subsets $U \subseteq V(G)$ to $W \subseteq V(H)$. Since $\alpha$ and $\beta$ are extensions of $\pi$, by definition we have $\alpha (x) = \pi (x)$ and $\beta (x) = \pi (x)$ for all $x \in U \subseteq V(G)$. Moreover *U* contains all reacting vertices induced by $\pi$. In symbols we have $V(\Gamma ^{\perp }(G[U], H[W], \pi )) \subseteq V(\Upsilon ^{\perp }(G[U], H[W], \pi )) = V(G[U]) = U$ and thus $\alpha (x) = \beta (x)$ holds in particular for all $x \in V(\Gamma ^{\perp }(G[U], H[W], \pi ))$. By definition we get $V(\Gamma ^{\perp }(G, H, \alpha )) = V(\Gamma ^{\perp }(G[U], H[W], \pi )) = V(\Gamma ^{\perp }(G, H, \beta ))$, since $\alpha$ and $\beta$ are stable extensions of $\pi$. Therefore $\Gamma ^{\perp }(G[U], H[W], \pi )$ contains all reacting vertices of $\Upsilon ^{\perp }(G, H, \alpha )$ and $\Upsilon ^{\perp }(G, H, \beta )$. Lemma 3 implies that $\alpha$ and $\beta$ coincide for all reacting vertices induced, in particular, by $\alpha$ for $G \longrightarrow H$, i.e., understood as reacting vertices $x \in V(G)$ and $\alpha (x) \in V(H)$. In addition, statement *(iii)* of Proposition 4 yields $\beta \in \operatorname {Iso}(\hat{G}_{\pi }, \hat{H}_{\pi })$, while statement *(ii)* ensures that $\hat{G}_{\pi } = \hat{G}_{\alpha }$ and $\hat{H}_{\pi } = \hat{H}_{\alpha }$. Thus $\beta$ is an isomorphism from the remainder graph $\hat{G}_{\alpha }$ to the remainder graph $\hat{H}_{\alpha }$. Proposition 1 therefore applies to $\alpha$ and $\beta$, and implies $\alpha \equiv \beta$. $\square$

Since all stable extensions of $\pi$ are equivalent, their ITS representations are isomorphic,

#### Corollary 2

Let $\pi$ be a good partial AAM for a balanced reaction $G \longrightarrow H$ and let $\alpha$ and $\beta$ be two stable extensions of $\pi$. Then $\Upsilon (G,H,\alpha ) \simeq \Upsilon (G,H,\beta )$.

A good partial atom map for a balanced reaction, therefore, uniquely determines the ITS graph of the full AAM for the reaction, up to graph isomorphism.

**Fig. 4:**
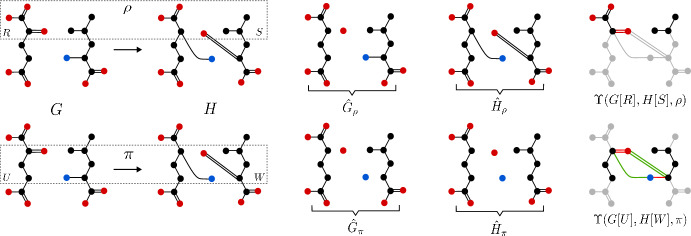
Two partial AAMs $\pi : U \rightarrow W$ and $\rho : R \rightarrow S$ over the reaction $G \longrightarrow H$ with full AAM $\alpha$ from Fig. 1. The reaction centers induced by these maps are depicted by the edges drawn in red and green in the respective partial ITS graphs $\Upsilon (G[U],H[W],\pi )$ and $\Upsilon (G[R], H[S],\rho )$. Since $\pi$ covers the reaction center induced by $\alpha$, then $\pi$ is a good partial AAM with stable extension $\alpha$, and equivalently the remainder graphs $\hat{G}_{\pi }$ and $\hat{H}_{\pi }$ are isomorphic. On the other hand, $\rho$ has no stable extensions, given that the corresponding remainder graphs $\hat{G}_{\rho }$ and $\hat{H}_{\rho }$ cannot be mapped to each other under an isomorphism

## Algorithms for completing good AAMs over balanced reactions

Conceptually, Definition 8 implies that the existence of a stable extension for a partial map $\pi$ over a reaction $G \longrightarrow H$ ensures that $\pi$ already provides all necessary information about the reaction mechanism of $G \longrightarrow H$. A bad partial AAM $\pi$, in contrast, fails to faithfully disclose the electron bookkeeping fundamental for understanding $G \longrightarrow H$. The characterization of stable extensions as isomorphisms of the reminder graphs $\hat{G}_{\pi }$ and $\hat{H}_{\pi }$ in Proposition 4, on the other hand, suggests to employ modified versions of algorithms for isomorphism search in order to test the existence of a stable extension, and thus the *goodness* of $\pi$, and to retrieve such extensions whenever they exist.

From a theoretical point of view, this *stable extension problem* can be solved efficiently for chemical graphs. To see this, we note that the bounded valency of atoms implies that graphs that represent molecules must have bounded degree. As an immediate consequence ITS graphs also have bounded degree. A classical result establishes that isomorphisms of graphs with bounded degree can be computed in polynomial time [27], although algorithms following this approach are not competitive in practice. For recent progress we refer to [28]. No implementations of these polynomial-time algorithms seem to have become available, however. Hence we have to resort to general purpose algorithms for graph isomorphism. This situation conveys, furthermore, the relevance of the uniqueness of stable extensions of good partial AAMs in Theorem 5, given that these depend, therefore, on the existence of one and only one of such constrained isomorphisms and are unambiguously determined by it.

We address the stable extension problem through three algorithmic approaches: (1) an *anchored* isomorphism search, (2) a *relabeling-and-isomorphism* strategy, and (3) an ILP approach. In [16] we devised said ILP formulation based on Lemma 1, and here we recapitulate it in order to benchmark it against the other methods. As shown later, the graph-based isomorphism searches perform better than the ILP in practice. Nonetheless, Theorem 5 implies that our methodologies are all **mathematically equivalent**, i.e., they return the same stable extension (up to equivalence of AAMs), whenever it exists.

**Anchored isomorphism tests.** Conceptually these constitute a variant within the VF2-family of algorithms designed for the (sub-)graph isomorphism problem. A detailed formulation of the VF2 algorithm can be found in [29]. This class of algorithms operates by progressively extending a candidate map for an isomorphism $\alpha : V(G) \rightarrow V(H)$ for arbitrary input graphs *G* and *H*. In each step, an ordered pair (*x*, *y*) called a *match*, with $x \in V(G)$ and $y \in V(H)$, is added to a collection $\mathcal {M}_{\alpha }$ portraying the vertex *y*, therefore, as the image of *x* under a prototype for $\alpha$. Later, if (*x*, *y*) is added to $\mathcal {M}_{\alpha }$, further candidate pairs to extend the map are then selected, either from the sets of *unmatched* neighbors of *x* and *y*, called *terminal* sets, or arbitrarily selected from the remaining unmatched vertices. This last case is specifically designed to process disconnected graphs, since it allows the selection of vertices from distinct components once the terminal sets are exhausted.

Though such progressive procedure was originally designed as a recursive traversal, more recent variations construct the match set $\mathcal {M}_{\alpha }$ in an iterative manner [30].

The extension of $\mathcal {M}_{\alpha }$ depends on evaluating the *syntactic feasibility* i.e., a one-to-one correspondence of the edges connecting *x* and *y* respectively, to the vertices already included in $\mathcal {M}_{\alpha }$, as well as the *semantic feasibility*. The latter evaluates that *x* and *y* have the same vertex-labels, and that corresponding edges incident with them have compatible edge-labels. In this way, whenever the VF2 finds that all available candidate pairs (*x*, *y*) are unfeasible for extending a current set of matches $\mathcal {M}_{\alpha }$, the algorithm backtracks by removing from $\mathcal {M}_{\alpha }$ the last added match, and then testing other alternative candidate pairs.

This progressive exploration behavior of available matches is ideal for our *anchored isomorphism* search, of which we include a high-level description in Algorithm 1. Given a partial map $\pi$, moreover, for a balanced reaction $G \longrightarrow H$, the collection $\mathcal {M}_{\pi }$ of pairs (*x*, *y*) with $\pi (x) = y$, actually constitutes an *initial state* for the set $\mathcal {M}_{\alpha }$ described before. In other words, since an isomorphism $\alpha$ between the reminder graphs $\hat{G}_{\pi }$ and $\hat{H}_{\pi }$, necessarily coincides with $\pi$ on all reacting vertices if it is a (stable) extension of $\pi$, the set $\mathcal {M}_{\pi }$, prepared in lines 8 to 10 of Algorithm 1, therefore acts as an *anchor* for seed the VF2 routine. This seed is then expanded by passing it to a further call to a regular VF2 routine in line 12.

By definition the reaction center of $G \longrightarrow H$ is composed of unmatchable edges, that is, the reaction edges in *G* cannot be matched by the VF2 with edges in *H*, and similarly, reaction edges in *H* will have no matching edge in *G*, i.e., these edges cannot satisfy either one or both feasibility tests of the VF2. Consequently, we remove the reaction edges in a preprocessing step in line 6 of our algorithm. We remove, moreover, all edges whose ends are both reacting vertices, simplifying the search even further whenever possible.

The remainder graphs $\hat{G}_{\pi }$ and $\hat{H}_{\pi }$ obtained after removing all reaction edges edges may be disconnected. As mentioned in Sect. , we are interested in applying Algorithm 1 only over balanced single-step reactions producing connected ITS graphs. Thus Lemma 4 implies that, even under such conditions, every connected component of $\hat{G}_{\pi }$ and $\hat{H}_{\pi }$ contains at least one reacting vertex. Hence all non-reacting vertices in $\hat{G}_{\pi }$ and $\hat{H}_{\pi }$ remain, in general, reachable during the progressive expansion with the VF2 through the terminal sets. This implies a reduction in complexity of the search space, in particular, when processing molecular graphs, by avoiding the exhaustive evaluation of trivial or non-informative matches.

**Algorithm 1:**
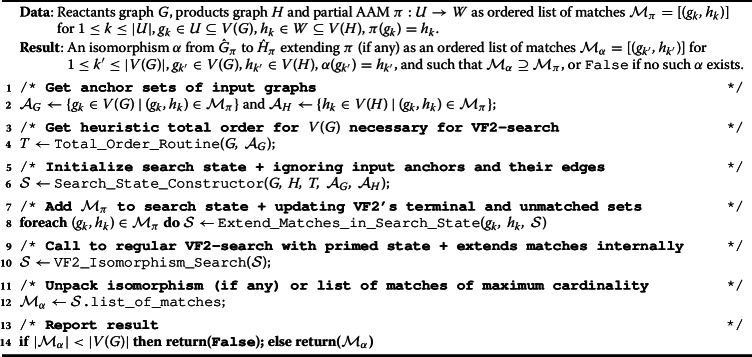
Anchored graph isomorphism search (VF2-variant)

Publicly available implementations of the VF2 algorithm, such as the one from the Python package NetworkX [31], do not provide default options to handle the initialization of a search state with an anchor map, as required by line 9 of Algorithm 1. We opted, therefore, to develop a custom implementation of this anchored search. For this we made use, in particular, of the Cython language [32], which allows the implementation of C and C++ data structures through a Python-like syntax, and facilitates the back-and-ford conversion of these data containers and data types, respectively, with Python native objects and types.

Our implementation is available as the routine search_stable_extension, inside the Python package GranMapache (**GRA**phs-and-**N**etworks **MAP**ping **A**pplications with **C**ython and **HE**uristics), in the repository: https://github.com/MarcosLaffitte/GranMapache, where we provide diverse functionalities to address problems related to mappings between graphs.

**Relabeling-and-isomorphism strategy.** Throughout this contribution, except for a few cases, e.g. for the definition of ITS graphs, we have considered vertex labels to represent atom types. Formally speaking, nonetheless, these labels embody the broader notion of *comparability classes* of vertices, i.e., two vertices are *comparable* with each other if and only if they have the same label.

Here we device a relabeling strategy, condensed in Algorithm 2, that offers an equivalent, but simpler alternative, to the anchored isomorphism approach described earlier. To illustrate this, consider a balanced reaction $G \longrightarrow H$ with a partial AAM $\pi : U \rightarrow W$ with $U \subseteq V(G)$ and $W \subseteq V(H)$, and four vertices $u, x \in V(G)$ and $w, y \in V(H)$. Suppose that $\pi (u) = w$ and $a_G(x) = a_H(y)$, but $x \notin U$ and $y \notin W$. Assume, moreover, that there exists a stable extension $\alpha$ of $\pi$ such that $\alpha (x) = y$. Thus, any algorithm capable of retrieving $\alpha$ as an isomorphism between the remainder graphs $\hat{G}_{\pi }$ and $\hat{H}_{\pi }$, has to (*i*) match again *u* and *w* without admitting for them any other matches and (*ii*) must be able to recognize *x* and *y* as comparable vertices. Clearly Algorithm 1 satisfies these conditions.

The same result, however, can be achieved by creating copies of $\hat{G}_{\pi }$ and $\hat{H}_{\pi }$ with new labeling functions, enforcing *comparability constraints* with these new labels. By slight abuse of notation we write $a'_G: V(G) \rightarrow \{0, 1, ..., |U|\} \times L_V$ for the labeling function on the copy of $\hat{G}_{\pi }$, and $a'_H: V(H) \rightarrow \{0, 1,..., |W|\} \times L_V$ for the copy of $\hat{H}_{\pi }$. This labels are described formally in lines 6 and 7 of Algorithm 2. With them, for the example above, the vertices matched by $\pi$ are now labeled by ordered pairs $a'_G(u) = (k, a_G(u)) = (k, a_H(w)) = a'_H(w)$ for a unique integer $k \in \{1, ..., |U|\}$, while remaining vertices are assigned a label $a'_G(x) = (0, a_G(x)) = (0, a_H(y)) = a'_H(y)$, thus satisfying as well conditions (*i*) and (*ii*) from before.

**Algorithm 2:**
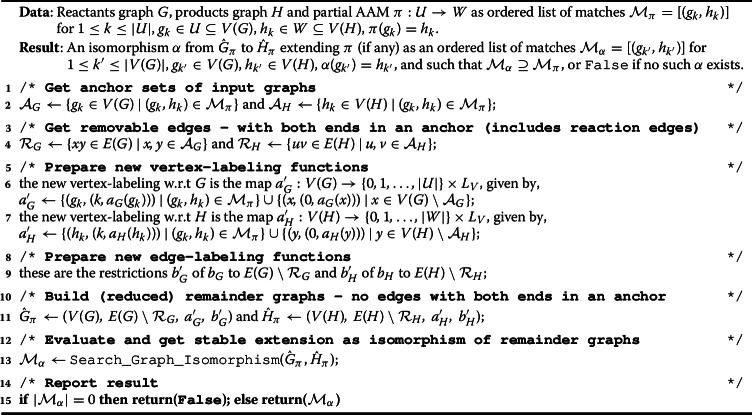
Special relabeling and graph isomorphism search

**Fig. 5:**
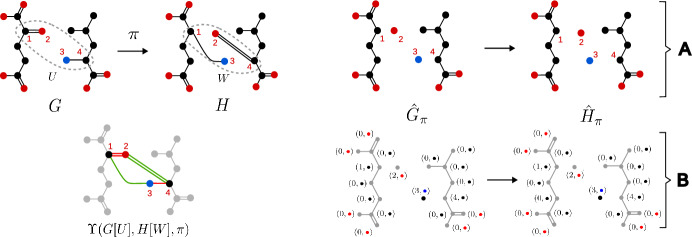
A good partial AAM $\pi$ for the reaction in Fig. 1 with full AAM $\alpha$. **a** shows the remainder graphs w.r.t $\pi$, and **b** their respective computational representation used for Algorithm 2. The new (pairs of) labels assigned to the remainder graphs facilitate the conservation of $\pi$ as an *anchor* during the search for $\alpha$, while allowing the comparison of the vertices not being mapped by $\pi$

Once the new labeling functions are built, we only need to create the copies of $\hat{G}_{\pi }$ and $\hat{H}_{\pi }$ as stated in line 11 of Algorithm 2, and finally run for these graphs an arbitrary isomorphism routine handling labeled graphs, as in line 13, in order to recover the stable extension $\alpha$. An example of the application of this algorithm is shown in Fig. 5.

We implemented this method in Python, making use of the NetworkX [31] package for the handling of graphs and their labeling functions. This implementation is also available in the repository GranMapache, in a directory dedicated to examples of usage of our functionalities:

https://github.com/MarcosLaffitte/GranMapache/tree/main/examples/Stable_Extensions.

**Integer Linerar Programming formulation.** Finally, Algorithm 3 describes, as a pipeline, the formulation of the Integer Linear Programming (ILP) search for stable extensions, that we originally proposed in [16]. For this we made use of the CBC solver 2.10.3 [33], made in C++ and callable from Python.

**Algorithm 3:**
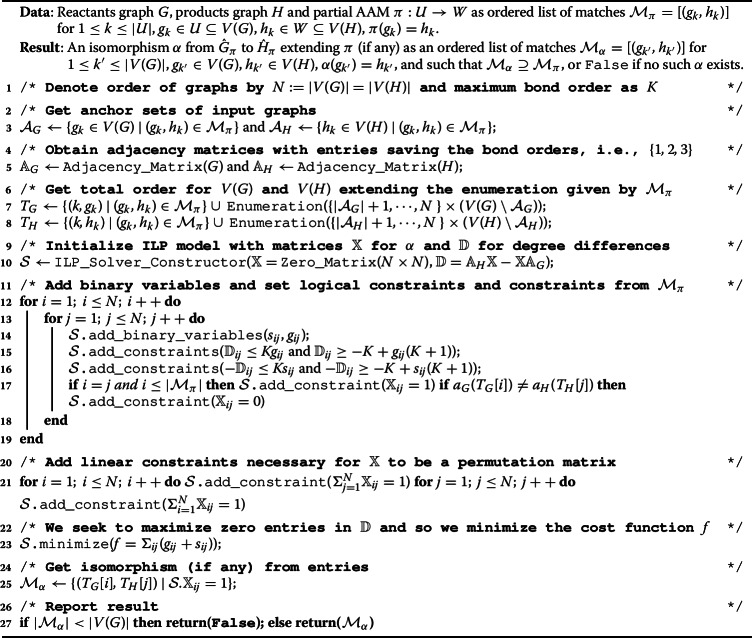
Isomorphism of remainder graphs with ILP

## Meta- and co-extension of AAMs and the hydrogen atom mapping problem (HAMP)

Reaction data are often unbalanced not only due to reactants or products that are not reported in database entries, but are also incomplete due to missing parts within the reactant or product molecules themselves. Most importantly, hydrogen atoms are regularly omitted from structural formulae. From a chemist’s perspective, these are often implicit, e.g. complementing the valency of carbon atoms to 4. The lack of explicit information on hydrogens, however, implies that AAMs for many reactions, and therefore their mechanisms, are incomplete.

We have been concerned with this complication in previous work as part of the applications of the formalism of partial AAMs [16]. Here we explore its connection to the problem of extending such partial data. More precisely, we consider the extension of AAMs specifically to reactions involving molecules which have actual *unrepresented* parts. From a theoretical perspective, we will not make the assumption that only hydrogen atoms are affected, which leads us to the concepts of *meta-extension* and *co-extension* of AAMs. From a practical point of view, by far the most common case concerns hydrogen atoms that are not represented explicitly, and hence are also missing in the reaction center. We therefore refer to the problem of extending AAMs to unrepresented hydrogen atoms as the *Hydrogen Atom Mapping Problem* (HAMP). Figure 13 in Sect. 10 shows such a partially mapped chemical reactions and its *hydrogen-extensions* in detail.

It is worth noting that other plausible applications of these concepts do exist. For example, a variant of the HAMP arises when retrieving AAMs from KEGG RCLASS data, because these usually encode only part of the AAM [15]. Theorem 8 at the end of this section, moreover, will allow us to study the extension of AAMs within the context of the HAMP, based on certain conditions about the vertex labels of the involved molecules. Before proceeding to such particular scenario, we consider first the general case of balanced reactions whose molecules have missing parts.

### Definition 9

Let $\alpha$ be a full AAM for a balanced reaction $G \longrightarrow H$. Consider a balanced reaction $\widetilde{G} \longrightarrow \widetilde{H}$ where $\widetilde{G}$ contains *G* and $\widetilde{H}$ contains *H*, respectively, as proper vertex-induced subgraphs. We say that $\widetilde{G} \longrightarrow \widetilde{H}$ is $\alpha$-*explained* by $G \longrightarrow H$, if there is a full AAM $\widetilde{\alpha }$ for $\widetilde{G} \longrightarrow \widetilde{H}$ that is also a stable extension of $\alpha$ in this reaction, i.e., such that *(i)* $\widetilde{\alpha }(x) = \alpha (x)$ for all $x \in V(G) \subsetneq V(\widetilde{G})$ and *(ii)* $\Gamma ^{\perp }(\widetilde{G}, \widetilde{H}, \widetilde{\alpha }) = \Gamma ^{\perp }(\widetilde{G},\widetilde{H}, \alpha )$.

We say, moreover, that an arbitrary AAM $\mu$ of $\widetilde{G} \longrightarrow \widetilde{H}$ is a *meta-extension* of $\alpha$ into $\widetilde{G} \longrightarrow \widetilde{H}$ if condition *(i)* of Definition 9 is satisfied, i.e., $\mu (x) = \alpha (x)$ for all $x \in V(G) \subsetneq V(\widetilde{G})$. Accordingly, $\mu$ will be called a *stable meta-extension* of $\alpha$ into $\widetilde{G} \longrightarrow \widetilde{H}$ if it also conserves the reaction center implied by $\alpha$ for these reactions, i.e., $\Gamma ^{\perp }(\widetilde{G}, \widetilde{H}, \mu ) = \Gamma ^{\perp }(\widetilde{G},\widetilde{H}, \alpha ) = \Gamma ^{\perp }(G, H, \alpha )$.

The reaction $G \longrightarrow H$ with AAM $\alpha$ that is to be extended into a reaction $\widetilde{G} \longrightarrow \widetilde{H}$, therefore is a formal representation for all the molecules with missing parts, while these parts themselves are determined, in turn, by the vertices and edges of the graphs $\widetilde{G}$ and $\widetilde{H}$ not already belonging to, respectively, *G* and *H*. We start with the simple observation that *good* partial AAMs are equivalently described by *stable* meta-extensions. More precisely, from Definitions 8 and 9 it follows

### Proposition 6

Let $G \longrightarrow H$ be a balanced reaction and let $\pi : U \rightarrow W$, with $U \subseteq V(G)$ and $W \subseteq V(H)$, be a proper partial AAM for $G \longrightarrow H$. Then, $\pi$ is a good partial AAM for $G \longrightarrow H$ with stable extension $\alpha$, if and only if, $G \longrightarrow H$ is $\pi$-explained by $G[U] \longrightarrow H[W]$ with stable meta-extension $\alpha$.

**Fig. 6:**
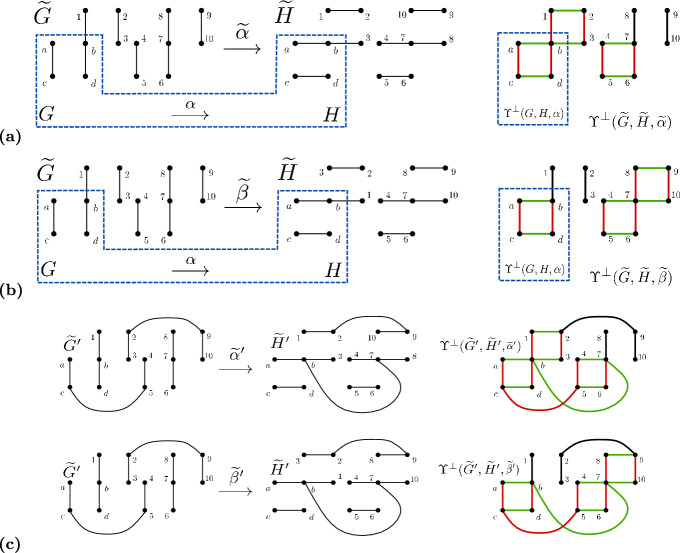
The isomorphism of ITS graphs, in the general case, does not imply the co-extension relation between AAMs extending a common anchor map. Here, frames **a** and **b** show an AAM $\alpha$ for a reaction $G \longrightarrow H$ being extended by distinct AAMs, $\widetilde{\alpha }$ in **(a)** and $\widetilde{\beta }$ in **(b)**, for a balanced reaction $\widetilde{G} \longrightarrow \widetilde{H}$. Vertices being matched together by an AAM are labeled in the figure with the same index, taken from the sets $\{a, b, c, d\}$ for vertices matched by $\alpha$, and from $\{1,..., 10\}$ for those matched by $\widetilde{\alpha }$ or $\widetilde{\beta }$ but not $\alpha$, i.e., for $\mu \in \{\alpha , \widetilde{\alpha }, \widetilde{\beta }\}$ we assign the same index to vertices *x* and $\mu (x)$ in their respective domain and image graphs. Since the ITS graphs $\Upsilon ^{\perp }(\widetilde{G}, \widetilde{H}, \widetilde{\alpha })$ and $\Upsilon ^{\perp }(\widetilde{G}, \widetilde{H}, \widetilde{\beta })$ are isomorphic, then $\widetilde{\alpha }$ and $\widetilde{\beta }$ are equivalent AAMs for $\widetilde{G} \longrightarrow \widetilde{H}$. The small differences between them, nonetheless, make impossible for them to be $\alpha$-co-extenders. Specifically, any isomorphism extending an automorphism of $\Upsilon ^{\perp }(G, H, \alpha )$ should contain a permutation of the set *V*(*G*) as a sub-map. Establishing different matches for the vertices with indices $\{1, 3, 8, 10\}$, however, limits the possible correspondence of *V*(*G*) under any isomorphism to subsets disjoint from *V*(*G*) itself. Lastly, subfigure **(c)** shows a similar scenario w.r.t. another reaction $\widetilde{G}' \longrightarrow \widetilde{H}'$, with AAMs $\widetilde{\alpha }'$ and $\widetilde{\beta }'$ also extending $\alpha$. The respective ITS graphs are also isomorphic, but these AAMs fail again to be $\alpha$-co-extenders. This example shows, moreover, that this situation is not exclusive of disconnected ITS graphs, and equally not limited to ITS graphs with disconnected reaction centers

Figure 6 suggests that the framework of meta-extensions offers, on the one hand, a conceptual advantage in that the reaction $\widetilde{G} \longrightarrow \widetilde{H}$ is not necessarily fixed *a priori*, making it possible to speak of extensions of AAMs for $G \longrightarrow H$ into different reactions. On the other hand, and more importantly, Theorem 8 suggests that the formalism of meta-extensions provides a promising avenue towards studying the largely unexplored mathematical properties of *bad* partial AAMs.

Before fully delving into the analysis of *non-stable* meta-extensions as representations of bad partial AAMs, we consider a result on the uniqueness of the stable meta-extensions, analogous to the uniqueness of the extension of good partial AAMs proved earlier in Theorem 5. In other words, from Proposition 6 it follows that any stable meta-extension of an AAM $\alpha$ into a reaction $\widetilde{G} \longrightarrow \widetilde{H}$, can be expressed simply as an stable extension when thinking of $\alpha$ as a (good) partial AAM of $\widetilde{G} \longrightarrow \widetilde{H}$. Therefore, Theorem 5 and Corollary 2 immediately imply that:

### Corollary 3

Let $G \longrightarrow H$ be a balanced reaction with AAM $\alpha$. Suppose, moreover, that $G \longrightarrow H$ $\alpha$-explains a balanced reaction $\widetilde{G} \longrightarrow \widetilde{H}$ and let $\widetilde{\alpha }$ and $\widetilde{\beta }$ be two stable meta-extensions of $\alpha$ into $\widetilde{G} \longrightarrow \widetilde{H}$. Then $\widetilde{\alpha } \equiv \widetilde{\beta }$ and, equivalently, $\Upsilon (\widetilde{G}, \widetilde{H}, \widetilde{\alpha }) \simeq \Upsilon (\widetilde{G}, \widetilde{H}, \widetilde{\beta })$.

The situation becomes more complicated when we consider an AAM $\alpha$ for $G \longrightarrow H$ for which all meta-extensions into $\widetilde{G} \longrightarrow \widetilde{H}$ are non-stable, i.e., when $\alpha$ is not a good partial AAM for $\widetilde{G} \longrightarrow \widetilde{H}$. Two distinct meta-extensions $\mu$ and $\mu '$

may then produce non-isomorphic ITS graphs $\Upsilon (\widetilde{G}, \widetilde{H}, \mu )$ and $\Upsilon (\widetilde{G}, \widetilde{H}, \mu ')$. As a consequence, $\mu$ and $\mu '$ may be non-equivalent AAMs.

We shall see below, however, that if $\alpha$ is incomplete, i.e., not covering the reaction center of $\widetilde{G} \longrightarrow \widetilde{H}$, it is still possible, under certain conditions, to make use of the automorphisms of the ITS graph $\Upsilon (G, H, \alpha )$ in order to reduce the computational complexity of testing the equivalence of different AAMs for $\widetilde{G} \longrightarrow \widetilde{H}$ that extend $\alpha$. Such automorphisms then provide an *anchor* for isomorphism algorithms. In the remainder of this section we formalize this idea and derive Theorem 8 which serves as a basis for Algorithm 4 to address the Hydrogen Atom Mapping Problem.

### Definition 10

Let $\alpha$ be a full AAM for a balanced reaction $G \longrightarrow H$ and let $\Upsilon ^{\perp }_{\alpha } :=\Upsilon ^{\perp }(G, H, \alpha )$ denote its corresponding (canonical) ITS graph. Consider a balanced reaction $\widetilde{G} \longrightarrow \widetilde{H}$ where $\widetilde{G}$ and $\widetilde{H}$ contain, respectively, *G* and *H* as proper induced subgraphs. Let $\widetilde{\alpha }$ and $\widetilde{\beta }$ be two (not necessarily stable) meta-extensions of $\alpha$ into $\widetilde{G} \longrightarrow \widetilde{H}$, and let $\Upsilon ^{\perp }_{\widetilde{\alpha }} :=\Upsilon ^{\perp }(\widetilde{G}, \widetilde{H}, \widetilde{\alpha })$ and $\Upsilon ^{\perp }_{\widetilde{\beta }} :=\Upsilon ^{\perp }(\widetilde{G}, \widetilde{H}, \widetilde{\beta })$ denote their respective ITS graphs. We say that $\widetilde{\alpha }$ is an $\alpha$-*co-extender* of $\widetilde{\beta }$, if there exists an automorphism $\varphi \in \operatorname {Aut}(\Upsilon ^{\perp }_{\alpha })$ that can be extended into an isomorphism $\widetilde{\varphi }$ from $\Upsilon ^{\perp }_{\widetilde{\alpha }}$ to $\Upsilon ^{\perp }_{\widetilde{\beta }}$, i.e., if there is $\widetilde{\varphi } \in \operatorname {Iso}(\Upsilon ^{\perp }_{\widetilde{\alpha }}, \Upsilon ^{\perp }_{\widetilde{\beta }})$ such that $\varphi (x) = \widetilde{\varphi }(x)$ for all $x \in V(G)$.

It is important to note that $V(\Upsilon ^{\perp }_{\alpha }) = V(\Upsilon ^{\perp }_{\widetilde{\alpha }}) = V(\Upsilon ^{\perp }_{\widetilde{\beta }}) = V(G)$, and thus the extension of the automorphism $\varphi \in \operatorname {Aut}(\Upsilon ^{\perp }_{\alpha })$ into an isomorphism $\widetilde{\varphi } \in \operatorname {Iso}(\Upsilon ^{\perp }_{\widetilde{\alpha }}, \Upsilon ^{\perp }_{\widetilde{\beta }})$, if any, is well defined. Indeed, this definition is motivated in the first place by the fact that $\Upsilon ^{\perp }_{\alpha } = \Upsilon ^{\perp }_{\widetilde{\alpha }}[V(G)] = \Upsilon ^{\perp }_{\widetilde{\beta }}[V(G)]$ and thus $\operatorname {Iso}(\Upsilon ^{\perp }_{\widetilde{\alpha }}[V(G)], \Upsilon ^{\perp }_{\widetilde{\beta }}[V(G)]) = \operatorname {Aut}(\Upsilon ^{\perp }_{\alpha })$. Next we show that the collection of all the AAMs that are co-extenders of a same given anchor map in fact forms an equivalence class.

### Proposition 7

The $\alpha$-co-extension between meta-extensions of a given AAM $\alpha$ is an equivalence relation.

### Proof

We follow the notation established in Definition 10. Reflexivity of this relation follows directly from the existence of both identity automorphisms, one for $\Upsilon ^{\perp }_{\alpha }$, and the other for $\Upsilon ^{\perp }_{\widetilde{\alpha }}$. That is, for the identity map $i_G: V(G) \rightarrow V(G)$, for which it holds $i_G \in \operatorname {Aut}(\Upsilon ^{\perp }_{\alpha })$, there is always the identity map $i_{\widetilde{G}}: V(\widetilde{G}) \rightarrow V(\widetilde{G})$ for which we have $i_{\widetilde{G}} \in \operatorname {Aut}(\Upsilon ^{\perp }_{\widetilde{\alpha }}) = \operatorname {Iso}(\Upsilon ^{\perp }_{\widetilde{\alpha }}, \Upsilon ^{\perp }_{\widetilde{\alpha }})$, and given that $i_{\widetilde{G}}(x) = x$ for all $x \in V(\widetilde{G})$, then in particular $i_G(x) = x = i_{\widetilde{G}}(x)$ for all $x \in V(G)$. Similarly, symmetry of the relation follows from the existence of the respective inverse bijections. To see this suppose first that $\widetilde{\alpha }$ is an $\alpha$-co-extender of $\widetilde{\beta }$, with $\varphi$ and $\widetilde{\varphi }$ being the required mappings satisfying **(P1)** $\varphi (x) = \widetilde{\varphi }(x)$ for all $x \in V(G)$. Then, note that for any $\varphi \in \operatorname {Aut}(\Upsilon ^{\perp }_{\alpha })$, we also have $\varphi ^{-1} \in \operatorname {Aut}(\Upsilon ^{\perp }_{\alpha })$, and in the same way for every $\widetilde{\varphi } \in \operatorname {Iso}(\Upsilon ^{\perp }_{\widetilde{\alpha }}, \Upsilon ^{\perp }_{\widetilde{\beta }})$ we always have $\widetilde{\varphi }^{-1} \in \operatorname {Iso}(\Upsilon ^{\perp }_{\widetilde{\beta }}, \Upsilon ^{\perp }_{\widetilde{\alpha }})$. In this way, by setting $y :=\varphi (x)$, for which clearly $y \in V(G)$, we get $\varphi ^{-1}(y) = x$, and thus **(P1)** rewritten as $\widetilde{\varphi }^{-1}(\varphi (x)) = x$, now yields $\widetilde{\varphi }^{-1}(y) = \varphi ^{-1}(y)$ for all $y \in V(G)$, meaning that $\varphi ^{-1}$ can be extended into $\widetilde{\varphi }^{-1}$ and thus $\widetilde{\beta }$ is also an $\alpha$-co-extender of $\widetilde{\alpha }$. Lastly, to prove transitivity we consider an additional meta-extension $\widetilde{\gamma }$ of $\alpha$. Suppose that $\widetilde{\alpha }$ is an $\alpha$-co-extender of $\widetilde{\beta }$, and respectively that $\widetilde{\beta }$ is an $\alpha$-co-extender of $\widetilde{\gamma }$. This means that there exists an automorphism $\varphi \in \operatorname {Aut}(\Upsilon ^{\perp }_{\alpha })$ for which there is an isomorphism $\widetilde{\varphi } \in \operatorname {Iso}(\Upsilon ^{\perp }_{\widetilde{\alpha }}, \Upsilon ^{\perp }_{\widetilde{\beta }})$ such that **(P2)** $\varphi (x) = \widetilde{\varphi }(x)$ for all $x \in V(G)$, and at the same time there exists an automorphism $\psi \in \operatorname {Aut}(\Upsilon ^{\perp }_{\alpha })$ for which there is an isomorphism $\widetilde{\psi } \in \operatorname {Iso}(\Upsilon ^{\perp }_{\widetilde{\beta }}, \Upsilon ^{\perp }_{\widetilde{\gamma }})$ such that **(P3)** $\psi (x) = \widetilde{\psi }(x)$ for all $x \in V(G)$. Since the composition of automorphisms (respectively of isomorphisms) is again an automorphism (resp. isomorphism), we see that $\psi \varphi \in \operatorname {Aut}(\Upsilon ^{\perp }_{\alpha })$ and that $\widetilde{\psi }\widetilde{\varphi } \in \operatorname {Iso}(\Upsilon ^{\perp }_{\widetilde{\alpha }}, \Upsilon ^{\perp }_{\widetilde{\gamma }})$. Then, by an application of **(P2)** followed by an application of **(P3)** we get that $\widetilde{\psi }\widetilde{\varphi }(x) = \widetilde{\psi }(\widetilde{\varphi }(x)) = \widetilde{\psi }(\varphi (x)) = \psi (\varphi (x)) = \psi \varphi (x)$ holds again for every $x \in V(G)$, implying that the $\alpha$-co-extension between meta-extensions of $\alpha$ is also symmetric, and therefore an equivalence relation. $\square$

Intuitively speaking, therefore, we are interested in using the information provided by $\alpha$, and contained in $\operatorname {Aut}(\Upsilon ^{\perp }_{\alpha })$, to simplify the computational task of identifying those meta-extensions of $\alpha$ into $\widetilde{G} \longrightarrow \widetilde{H}$ which happen to be equivalent AAMs with one another, i.e., if $\widetilde{\alpha }$ and $\widetilde{\beta }$ are $\alpha$-co-extenders, and thus the isomorphism implied by Definition 10 exists, then clearly by Proposition 2 it follows that $\widetilde{\alpha } \equiv \widetilde{\beta }$. In order to use this property computationally, however, we need to understand under which conditions the converse statement is true.

More precisely, we ask whether the isomorphism of the ITS graphs $\Upsilon ^{\perp }_{\widetilde{\alpha }}$ and $\Upsilon ^{\perp }_{\widetilde{\beta }}$ is sufficient to imply that $\widetilde{\alpha }$ is an $\alpha$-co-extender of $\widetilde{\beta }$. Figure 6 shows that this is not true in general, i.e., there are equivalent AAMs $\widetilde{\alpha }$ and $\widetilde{\beta }$ that are not co-extenders of a given anchor map $\alpha$, even if they are equivalent as AAMs and are indeed extensions of $\alpha$. The AAM $\alpha$ therefore does not always contain enough information to explain a reaction with an AAM taken from the equivalence class of $\widetilde{\alpha }$ and $\widetilde{\beta }$. Theorem 8 nevertheless identifies conditions under which the co-extension relation is characterized by the isomorphism of ITS graphs.

### Theorem 8

Let $\alpha$ be a full AAM for a balanced reaction $G \longrightarrow H$ and let $\widetilde{\alpha }$ and $\widetilde{\beta }$ be two (not necessarily stable) meta-extensions of $\alpha$ into a balanced reaction $\widetilde{G} \longrightarrow \widetilde{H}$. Then, $\widetilde{\alpha }$ and $\widetilde{\beta }$ are $\alpha$-co-extenders, if and only if, there exists an isomorphism $\widetilde{\varphi }$ from $\Upsilon ^{\perp }(\widetilde{G}, \widetilde{H}, \widetilde{\alpha })$ to $\Upsilon ^{\perp }(\widetilde{G}, \widetilde{H}, \widetilde{\beta })$ such that $\widetilde{\varphi }(V(G)) = V(G)$.

### Proof

Following the notation from Definition 10, suppose first that $\widetilde{\alpha }$ and $\widetilde{\beta }$ are $\alpha$-co-extenders, and let $\widetilde{\varphi } \in \operatorname {Iso}(\Upsilon ^{\perp }_{\widetilde{\alpha }}, \Upsilon ^{\perp }_{\widetilde{\beta }})$ be the required isomorphism extending the automorphism $\varphi \in \operatorname {Aut}(\Upsilon ^{\perp }_{\alpha })$ implied by the definition. Since $\varphi (x)$ is a bijection from *V*(*G*) to itself, and given that $\widetilde{\varphi }$ is also a bijection that extends $\varphi$, we immediately see that $x \in \widetilde{\varphi }(V(G)) = \varphi (V(G))$ if and only if $x \in V(G)$, i.e., $\widetilde{\varphi }(V(G)) = V(G)$, as required.

For the converse, assume that the isomorphism $\widetilde{\varphi } \in \operatorname {Iso}(\Upsilon ^{\perp }_{\widetilde{\alpha }}, \Upsilon ^{\perp }_{\widetilde{\beta }})$, such that $\widetilde{\varphi }(V(G)) = V(G)$, is given. Note that for any two vertices $x, y \in V(\widetilde{G})$ it holds $xy \in E(\Upsilon ^{\perp }_{\widetilde{\alpha }})$ if and only if $\widetilde{\varphi }(x)\widetilde{\varphi }(y) \in E(\Upsilon ^{\perp }_{\widetilde{\beta }})$, while $\widetilde{\varphi }$ preserves, additionally, vertex and edge labels in these graphs. Thus this is true, in particular, for vertices $x, y \in V(G) \subseteq V(\widetilde{G})$. But since both $\widetilde{\alpha }$ and $\widetilde{\beta }$ extend $\alpha$ we have $\Upsilon ^{\perp }_{\alpha } = \Upsilon ^{\perp }_{\widetilde{\alpha }}[V(G)] = \Upsilon ^{\perp }_{\widetilde{\beta }}[V(G)]$. In this way, by letting $\varphi$ denote the restriction of $\widetilde{\varphi }$ to *V*(*G*), we see that for any two vertices $x, y \in V(G) = V(\Upsilon ^{\perp }_{\alpha })$ it holds $xy \in E(\Upsilon ^{\perp }_{\alpha })$ if and only if $\widetilde{\varphi }(x)\widetilde{\varphi }(y) \in E(\Upsilon ^{\perp }_{\alpha })$, where $\varphi$ preserves again vertex and edge labels in $\Upsilon ^{\perp }_{\alpha }$ and thus $\varphi \in \operatorname {Aut}(\Upsilon ^{\perp }_{\alpha })$. Then $\widetilde{\varphi }$ and $\varphi$ satisfy Definition 10, and $\widetilde{\alpha }$ and $\widetilde{\beta }$ are therefore $\alpha$-co-extenders, completing the proof. $\square$

The key condition in Theorem 8 is that the isomorphism $\widetilde{\varphi }$ of the ITS graphs satisfies $\widetilde{\varphi }(V(G)) = V(G)$, i.e., that it *stabilizes* the subgraph of the ITS graph for which the AAM $\alpha$ is known at the outset. In other words, $\widetilde{\varphi }$ does not “mix” atoms that are explicitly represented in *G* and *H* with atoms that are only present in $\widetilde{G}$ and $\widetilde{H}$. This condition is satisfied, in particular, by those cases where $\widetilde{G}$ and $\widetilde{H}$ are molecular representations that explicitly display all hydrogen atoms, while *G* and *H* comprise only and all other non-hydrogen atoms.

Theorem 8 thus is applicable in particular to HAMP scenarios. More precisely, we consider a balanced reaction $\widetilde{G} \longrightarrow \widetilde{H}$ for which it is known, for example, based on expert- or ground-truth knowledge, that there are hydrogen atoms playing a part within its mechanism. We suppose, moreover, that those reacting hydrogen atoms, together with all other non-hydrogen atoms, are explicitly represented in the graphs $\widetilde{G}$ and $\widetilde{H}$, while the non-reacting hydrogen atoms are assumed to be present only through the valency of the other elements. This last aspect is of importance, in particular, to reduce the computational cost of producing an AAM for $\widetilde{G} \longrightarrow \widetilde{H}$, as well as for obtaining the automorphism group of the corresponding ITS graph.

Without loss of generality, we consider then an AAM $\alpha$ matching all the non-hydrogen atoms of $\widetilde{G}$ to the non-hydrogen atoms of $\widetilde{H}$. In other words, $\alpha$ is an AAM for the balanced reaction $G \longrightarrow H$ where *G* and *H* are the (proper) subgraphs of $\widetilde{G}$ and $\widetilde{H}$, resp., that are induced by the non-hydrogen vertices. In this scenario we assume, furthermore, that no prior full AAM for $\widetilde{G} \longrightarrow \widetilde{H}$ is given. In practice, some hydrogen atoms may be represented explicitly while others are omitted. This situation appear in particular in manually curated data, where we may assume that $\alpha$ is specified for the hydrogen atoms in *G* and *H*. We may then ensure that $\widetilde{\varphi }$ stabilizes *G* by distinguishing the hydrogens represented in *G* from those present only in $\widetilde{G}$ by means of a different label for the “unmatched hydrogens”.

A solution of the HAMP entails the enumeration and comparison of all possible meta-extensions of $\alpha$ into $\widetilde{G} \longrightarrow \widetilde{H}$. Since we assume that the hydrogen atoms represented in $\widetilde{G} \longrightarrow \widetilde{H}$, but not in $G \longrightarrow H$, and that at least some hydrogen atoms are part of the reaction center of $\widetilde{G} \longrightarrow \widetilde{H}$, then none of the meta-extensions are stable. Moreover, if the reaction $\widetilde{G} \longrightarrow \widetilde{H}$ has $N_h \ge 2$ “unmatched” reacting hydrogen atoms, there are $N_h!$ distinct meta-extensions to consider, since there are $N_h!$ bijections between these hydrogen atoms.

Similar to the general atom-to-atom mapping problem, chemical knowledge is also required to identify a chemically-correct hydrogen-extension. To the best of our knowledge, no complete and reliable solution to this restricted AAM problem is available. Nevertheless, our framework can assist by comparing the ITS graphs corresponding to the possible hydrogen-extensions and providing a human curator or computational evaluation tool with single representatives of the equivalence classes of $\alpha$-co-extensions. The corresponding computational workflow is summarized in Algorithm 4, whose implementation and benchmarking are discussed Sect. 10 below.

**Algorithm 4:**
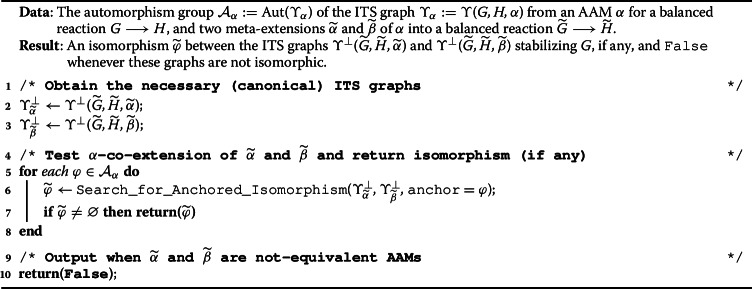
Test for Anchored Isomorphism $\widetilde{\varphi }$ Stabilizing *G*

Algorithm 4 retains a single representative of each isomorphism class of the ITS graph $\Upsilon ^{\perp }_{\widetilde{\alpha }}$, i.e., of each isomorphism class of hydrogen-extension $\widetilde{\alpha }$ to avoid redundant isomorphism checks. A further reduction of the effort is achieved by pre-computing the automorphism group of $\Upsilon ^{\perp }(G, H, \alpha )$ only once, and then using these as anchors, i.e., initial states, of the isomorphism search of ITS graphs. The computation of the the “anchor isomorphism” in Line 6 of Algorithm 4 can be performed by any subroutines presented in Algorithms 1, 2 or 3, which are implemented in such a way that they are readily applicable not only to the remainder graphs but also to ITS graphs. In fact, since the anchor maps are already assumed to be automorphisms of the reaction center $\Gamma ^{\perp }(G, H, \alpha )$, these edges can be still safely removed from $\Upsilon ^{\perp }(\widetilde{G}, \widetilde{H}, \widetilde{\alpha })$ and $\Upsilon ^{\perp }(\widetilde{G}, \widetilde{H}, \widetilde{\beta })$ during the search, in analogous manner to the analysis of remainder graphs. More details, and an example of this search, are presented and discussed further in Sect. 10.

## Benchmarking, empirical results, and discussion

We evaluate the methodologies discussed in the previous sections, first testing the recovery of stable extensions as discussed in Sect. , and later evaluating the method presented in Sect. 27 for determining the co-extension of AAMs and its application to the Hydrogen Atom Mapping Problem.

The existence of stable extensions is evaluated on two sets of empirical data, one of real chemical reactions with partial AAMs covering exactly the ground-truth reacting vertices, and another set consisting of random graphs with AAMs inducing connected ITS graphs with connected reaction centers and an increasing number of nodes and edges. For this purpose four different implementations are evaluated: (**GM**) our custom anchored isomorphism search from our package GranMapache [34] implementing Algorithm 1, two relabeling-and-isomorphism variants implementing Algorithm 2 from which one (**RB**$_1$) uses the VF2 isomorphism function [35] from NetworkX and another (**RB**$_2$) uses a custom isomorphism function from GranMapache, and lastly (**ILP**) implementing the ILP formulation in Algorithm 3 from the package AAMutils introduced in previous work [16]. We identify, moreover, the number of isomorphism classes formed by the reaction centers of the real-life reactions, and determine the distribution of these classes of mechanisms.

Lastly, we evaluate the method described in Sect. 27 for determining the co-extension of AAMs. This analysis of co-extension in the context of the HAMP is also performed for a set of real chemical reactions and for randomly generated ITS graphs.

*Stable extensions over real chemical reactions*

The reactions were retrieved from USPTO_50K corpus of 50,016 reactions [36]. Each reaction was rebalanced with the tool SynRBL [37] and preprocessed with the SynTemp pipeline [14], which enforces ensemble consistency, resolves hydrogen ambiguity and validates that the partial maps are good. The preprocessing stage yield 39,732 rebalanced and consistent reactions with full AAMs, good partial AAMs derived from the full ones, and the corresponding ITS graphs. We process the mapped reactions in SMILES format, via another custom tool SynKit for the methods **GM**, **RB**$_1$ and **RB**$_2$, and with the AAMutils API [16] for the **ILP**.

The 39,732 reactions were processed five times with each of the four methods. Here we report the average running time of these procedures over this data set. From Theorem 5 and Corollary 2, moreover, it follows that all possible full AAMs recovered by our algorithms are to be equivalent and thus produce isomorphic ITS graphs. As a back-up test for our implementations, therefore, we corroborate the successful recovery of the full AAMs by means of the isomorphism of the initial and recovered ITS graphs. The graph-based methods **GM**, **RB**$_1$ and **RB**$_2$ were able to recover 100% of the ground AAMs of the 39,732 reactions, while the **ILP** retrieved the AAMs successfully in 99.48% of the reactions. The few ILP mismatches are attributable to discrepancies in the canonicalization SMILES during the conversion of the output of reaction-mapping tools to the graph representations used here.

**Fig. 7:**
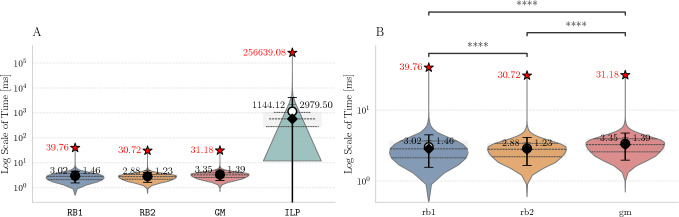
**a** Running times per reaction and per execution of the four methods. **b** Running time focused only on the graph-based methods **GM**, **RB**$_1$ and **RB**$_2$. Numerical values [ms] of average and maximum outlier are reported in black and red, respectively

Figure 7 below shows the distributions of the average running time per reaction for each method, and with respect to each of the five repetitions. The numerical values of the average running times per trial are summarized in Table 1.

**Table 1:** Average time to complete one partial atom mapping (mean ± std in ms)

Trial	GM (ms)	${RB_1}$ (ms)	${RB_2}$ (ms)	ILP (ms)
1	$3.39 \pm 1.44$	$3.07 \pm 1.54$	$2.92 \pm 1.30$	$1153.77 \pm 2983.54$
2	$3.33\pm 1.38$	$3.00 \pm 1.45$	$2.87 \pm 1.22$	$1142.43 \pm 2978.49$
3	$3.36 \pm 1.40$	$3.01 \pm 1.46$	$2.87 \pm 1.23$	$1139.71 \pm 2979.89$
4	$3.35 \pm 1.38$	$3.02 \pm 1.49$	$2.87 \pm 1.21$	$1135.71 \pm 2967.40$
5	$3.34 \pm 1.39$	$3.00 \pm 1.45$	$2.88 \pm 1.23$	$1148.98 \pm 2991.35$
Average	${3.36 \pm 1.39}$	${3.02 \pm 1.46}$	${2.88 \pm 1.22}$	${1144.12 \pm 2979.50}$

All benchmarks were run under Python 3.11 on a 12-core Intel Core i7-8700 @ 3.20 GHz, Fedora 37. The programs made for this analysis can be found in [38]

While the ILP average running time exceeds 1 second, all graph-based methods take only a few milliseconds ($p<0.05$). Among these, **RB**$_2$ is the fastest on average for processing the molecular graphs ($2.88\pm 1.22$ ms), followed by **RB**$_1$ ($3.02\pm 1.46$ ms) and **GM** ($3.36\pm 1.39$ ms). The small differences of 0.14 to 0.48 ms attained on this set of real molecular graphs, and disclosed by Fig. 7, are attributable to the time required by the custom implementations **RB**$_2$ and **GM** to convert native Python objects into C++ containers and vice versa, carried by the Cython functions on run time when called by other external Python scripts.

**Fig. 8:**
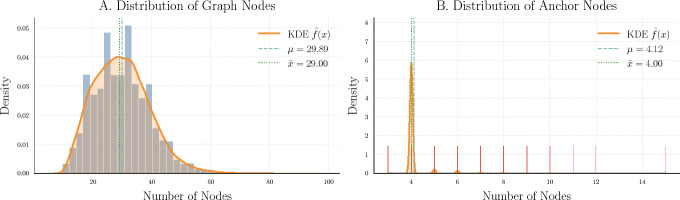
Histogram of number of vertices in molecular graphs (**a**) and in anchored graphs (**b**)

Tests for scalability were carried by analyzing the obtained running times while varying the number of vertices in the input molecular graphs. The distribution of the reactions with each number of vertices is shown in Fig. 8A.

**Fig. 9:**
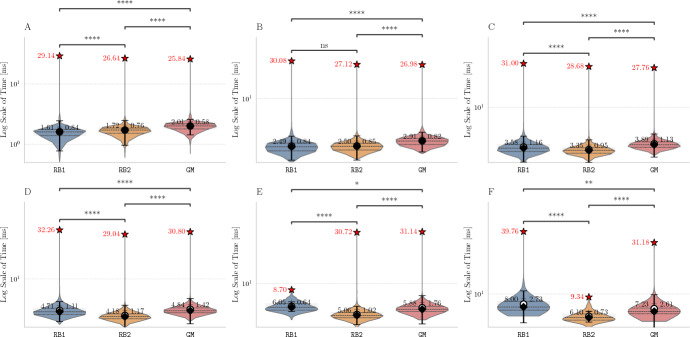
Running times of **RB**$_1$, **RB**$_2$, and **GM** as a function of ITS graph bins by number of vertices (top to bottom and left to right: less than 20, 20–30, 30–40, 40–50, 50–60, more than 60 vertices). **RB**$_1$ is faster on average for small graphs (<30 vertices), while **RB**$_2$ and **GM** exhibit better scalability on larger graphs, with a crossover at approximately 30 vertices

On the other hand, Fig. 9 shows that for small graphs (less than 30 vertices), **RB**$_1$ is faster than **RB**$_2$ and **GM**, while beyond the 30-vertices threshold **RB**$_2$ scales more favorably. Moreover, **GM** surpasses **RB**$_1$ on larger graphs, suggesting that the custom implementations **RB**$_2$ and **GM** offer an scalability advantage with respect to the amount of vertices in the input graphs. The small discrepancies in the running time of **RB**$_2$ and **GM** is further explained by the implementation of different heuristics for building the total order required by VF2-like approaches. The trial and evaluation of such heuristics is out of the scope of this contribution.

**Fig. 10:**
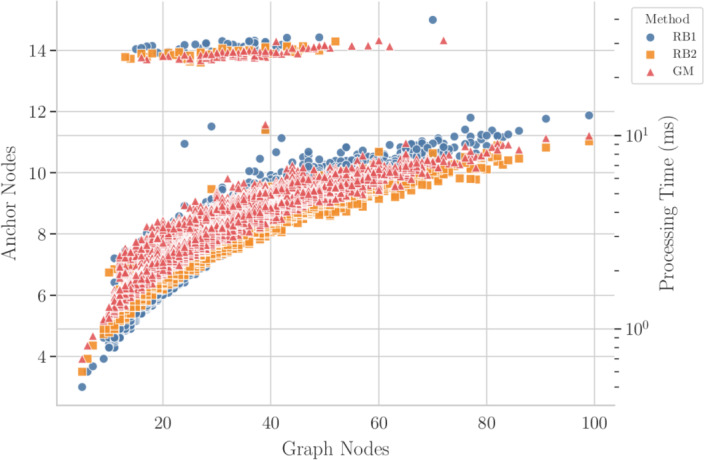
**Scatter plot projection.** Counts of graph vertex (x-axis) vs of reacting vertices (left y-axis) and running times (right y-axis [ms]) for the **GM**, **RB**$_1$, and **RB**$_2$ methods

Another scalability analysis was carried with respect to the proportion of reacting vertices vs total vertices in the ITS graph of each reaction. See the distribution of such proportions in Fig. 8B. The scatter plot in Fig. 10, together with Figs. 15 and 16 in Appendix A, suggest that all graph-based methods perform best for reactions with bigger proportions of reacting vertices from the total amount of vertices. This is expected since smaller proportions of reacting vertices lead to a bigger search space for the VF2-based approaches.

*Isomorphism classes of reaction centers from real chemical reactions*

The reaction centers inferred for the 39,732 real reactions where compared and clustered into (equivalence) classes of graph isomorphism with the SynKit suite. From this analysis we obtained 270 classes in total. The two most frequent of these consisted of 7,547 and 5,958 representatives, respectively, of $\sim 19\%$ and $\sim 15\%$ of the reactions. Mechanistically, the former of these corresponds to N-acylation and the latter to N-alkylation.

**Fig. 11:**
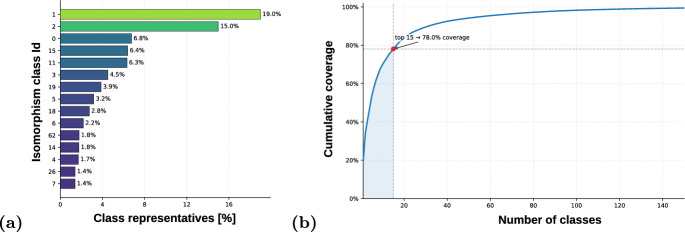
**a** Percentage share of the top 15 classes of reaction centers. **b** Cumulative coverage of the dataset when ranking the classes by frequency

Overall, the top 15 most frequent reaction center classes, moreover, cover together $\sim 78\%$ of the whole data set as shown in Fig. 11. Future work comprises the detail identification of the named reactions, i.e., of the reaction mechanism, that these classes represent, conveying a combined endeavor of manual and automatic analysis that is out of the scope of the current contribution.

*Stable extensions over random graphs*

We implemented the generation and analysis of random ITS graphs in Cython making use, in particular, of NetworkX [31]. All scripts for this analysis can be found in the GranMapache repository [34], and were run under Python 3.11 on an 8-core 11th Gen Intel Core i7-1165G7 @ 2.80GHz, Lenovo ThinkPad E15 Gen 2 with 16GB and Ubuntu 22.04.

For this analysis we produced connected ITS graphs with connected reaction centers. These graphs were built with an increasing amounts of edges outside the reaction center so as to test the performance of our methods over (simple) labeled graphs with varying density, i.e., with an increasing proportion of existing edges in the graph with respect to the theoretical maximum. Based on this we built 5 data sets of such graphs having each a different number of non-reacting vertices, specifically for 100, 125, 150, 175 and 200 nodes.

Within each data set we built 10 ITS graphs per each percentage-point for the percentage of edges outside the reaction center, starting at 3% and up to 97%. This leads to a total of 4,750 randomly generated ITS graphs conforming $\sim$1.6 GB of labeled graphs serialized and stored with the package Pickle [39] from Python. The interval 3–97 % was chosen under theoretical reasons for the graphs to be connected and for them to have the exact specified density. All the graphs were produced with a (randomly generated) connected reaction center of 15 vertices and 20 edges, in addition to the specified number of non-reacting vertices.

The pairs of labels needed for vertices and edges of these ITS graphs were chosen uniformly at random from a set of 6 integer labels for the vertices, and 3 labels for the edges. The labels in the reaction center, moreover, were produced by selecting the *source* of the (reaction) edge uniformly at random from the reactants graph, the products graph, or both of them. Finally, we tested the extension of the partial map covering exactly the reaction center.

**Fig. 12:**
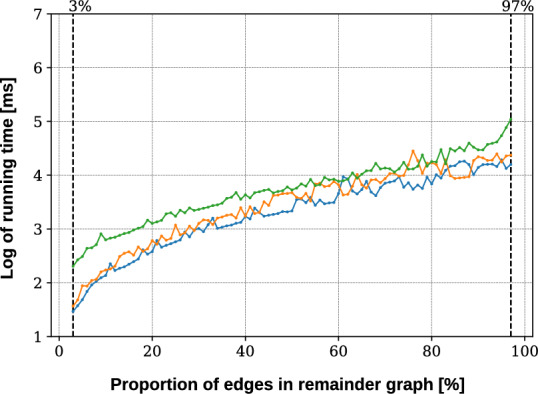
Average running times of different algorithms for stable extensions **RB**$_1$: green, custom **RB**$_2$: orange, and **GM**: blue) on ITS graphs with remainder graphs having 100 nodes and varying densities

For this we made use of the graph-based methods **RB**$_1$, **RB**$_2$ and **GM**, but omitted the **ILP** approach due to its comparably slower performance. All methods successfully extended the reaction center in all cases. The average running times for the analysis over the graphs with 100 non-reacting vertices are summarized in Fig. 12, while the results for graphs with 125–200 vertices are included in Fig. 17 in Appendix A. These show a consistent hierarchical performance, where **GM** completes the analysis faster, followed by **RB**$_2$, and then **RB**$_1$. This is consistent with the observations over real reactions, where molecules have at most 98 vertices and 108 edges and thus density $\le 2.28 \%$. This suggests again that the custom methods **GM** and **RB**$_2$ are appropriate for dealing with bigger graphs, while **RB**$_1$ proves to be comparably efficient for processing smaller and more sparse graphs.

*Co-extension and HAMP over real chemical reactions*

**Fig. 13:**
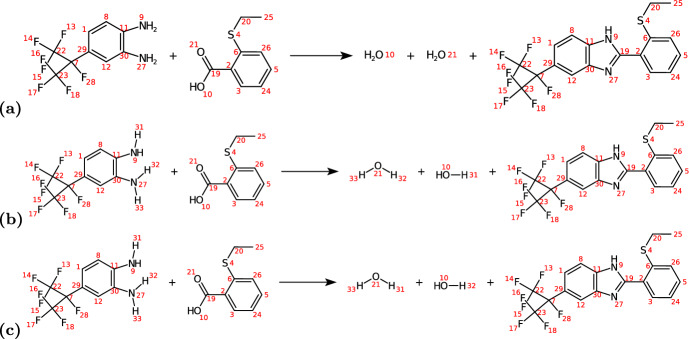
The indices in color red showcase **a** one base AAM and **b**–**c** two hydrogen-extensions for it, determined all for a *Phillips-Ladenburg condensation* reaction. Specifically, **a** shows a partially mapped (balanced) reaction with implicit hydrogens and with 30 non-hydrogen atoms, whose indices range from 1 to 30. Its hydrogen-extensions in **b** and **c** are two AAMs corresponding two distinct permutations of the hydrogen atoms now made explicit in the reaction with indices 31, 32 and 33

As discussed in Sect. 27, partial AAMs may omit hydrogens at the reaction center. To obtain a complete atom map, we enumerate all admissible hydrogen permutations and cluster the resulting maps to retain only unique hydrogen-completed extensions. As an example, Fig. 13 illustrates this for the *Phillips–Ladenburg condensation*: three hydrogen permutations collapse into two unique extensions in Fig. 13**b** and **c**, starting from the partially mapped reaction in Fig. 13**a**.

The task at hand, therefore, is to partition the ITS graphs of hydrogen-extensions into equivalence classes with respect to graph isomorphism. We benchmark the running-time of two strategies: (**Method A**) performs the iterative isomorphism test for pairs of ITS graphs representing each candidate extension, while clustering them into isomorphism classes. (**Method B**) first determines the automorphism group of the ITS graph without the explicitly unmatched hydrogens, and then applies Algorithm 4 to determine the co-extension of a given pair of ITS graphs for the hydrogen-extensions by using the automorphisms of the base ITS graph as anchors. The final step is the same iterative clustering of the reactions. This serves as a test of the practical feasibility of Algorithm 4 for the comparison of hydrogen-extensions in contrast to direct isomorphism testing. We emphasize, moreover, that neither method carries the exhaustive pairwise comparison of ITS graphs when clustering them, but rather this is done by comparing each ITS graph to only one of the subsequent representatives of each isomorphism class found throughout the iteration.

For each of these methods we consider three variants distinguished by isomorphism search routine presented in Sect. : Variant **AN-gm** uses the anchored isomorphism search of Algorithm 1, implemented in our custom toolkit GranMapache [34]. The two remaining variants **RB-gm** and **RB-nx** both use the relabeling strategy of Algorithm 2. While **RB-gm** uses the isomorphism search function from GranMapache, **RB-nx** employs the VF2 routine available in NetworkX [31].

For the broader analysis we identify, from the previously preprocessed subset of 39,732 reactions from USPTO_50K, those having at least 2 unmatched hydrogen atoms, since reactions with only 1 such atom can be trivially extended. This is done by comparing the *hydrogen-count* associated to all hetero- and carbon atoms. This information is readily available within the SMILES format of each reaction, and allows us to determine the exchange of hydrogen and protons. In particular, exchange with the medium is considered by retaining only those reactions explicitly representing the medium’s composition, e.g.  the inclusion of water molecules, thus allowing us to study reactions with a computational representation that is balanced up to hydrogen atoms. After this additional preprocessing step, the set of 39,732 reactions is reduced to a collection of 104 reactions, out of which 38 have only 2 unmatched hydrogens, 34 have 3, 22 have 4 and 10 have 5.

**Table 2:** Average running time for clustering ITS graphs corresponding to hydrogen extensions of real chemical reactions with a number $N_h = 2, 3, 4, 5$ unmatched hydrogen atoms (mean ± std in ms)

$N_h$	AN-gm	RB-gm	RB-nx
	Method A [ms]		
2	2.35 ± 0.90	2.41 ± 0.90	30.46 ± 161.00
3	10.81 ± 3.56	11.19 ± 3.67	14.18 ± 12.99
4	72.94 ± 41.99	77.55 ± 43.23	112.69 ± 124.94
5	973.43 ± 469.73	980.46 ± 486.19	661.28 ± 452.26
	Method B [ms]		
2	218.45 ± 1176.45	273.90 ± 1476.16	609.40 ± 3541.65
3	83.20 ± 198.30	97.72 ± 232.05	78.42 ± 168.80
4	1412.16 ± 4450.19	1652.07 ± 5208.70	1200.15 ± 3669.04
5	3986.38 ± 4350.32	4564.01 ± 5084.11	3130.38 ± 3138.05

The experimental running-times for the set of real reactions are summarized in Table 2 as a function of the number $N_h$ of unmatched hydrogen atoms. On average, **Method A** performs better than **Method B** on this data set.

Moreover, the variants **AN-gm** and **RB-gm** using our custom implementations, run faster that the variant **RB-nx** making use of the VF2 functionalities from NetworkX, except for $N_h = 5$. Both observations can be explained by the gradients shown in Fig. 21 of Appendix A. In particular Fig. 21b shows that the performance of variants like **AN-gm** is negatively affected by an increasing number of equivalence classes, which in turn is linked to a larger number of unmatched hydrogen atoms. As expected, **Method B** is affected most strongly be the number of automorphisms of the base ITS graph, since Algorithm 4 iterates over this automorphism group.

**Fig. 14:**
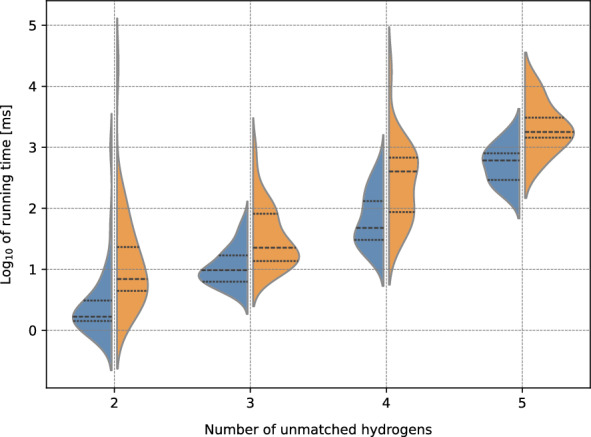
Violin plots for the mirrored distributions of the running time of **RB-nx** with respect to **Method A** in blue, and **Method B** in orange, for the analysis over real chemical reactions. The horizontal dotted lines indicate the quartiles of each distribution

**Fig. 15:**
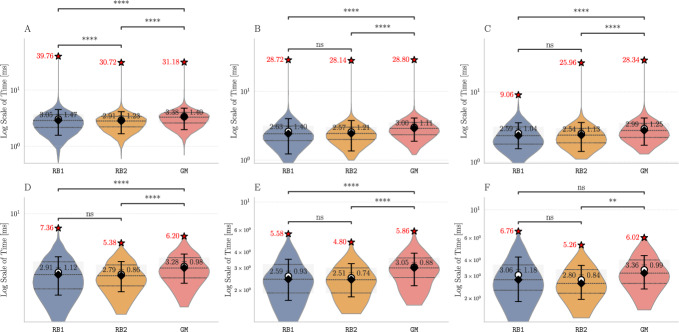
Running time comparison of **RB**$_1$, **RB**$_2$, and **GM** across six non-reacting vertex count-bins (top to bottom and left to right: less than 4, 4, 5, 6, 7, and at least 7 vertices)

**Fig. 16:**
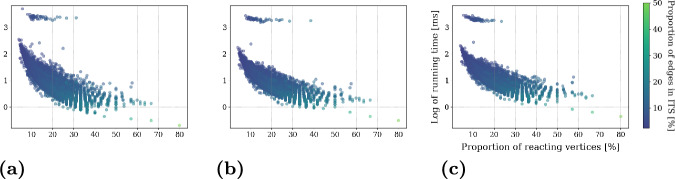
Scatter plots of running time against proportion of reacting vertices in real reactions for the graph based-methods **(a)** **RB**$_1$, **(b)** **RB**$_2$ and **(c)** **GM**. A smaller proportion of reacting vertices leads to a bigger search space for the VF2-based approaches and thus bigger running times

**Fig. 17:**
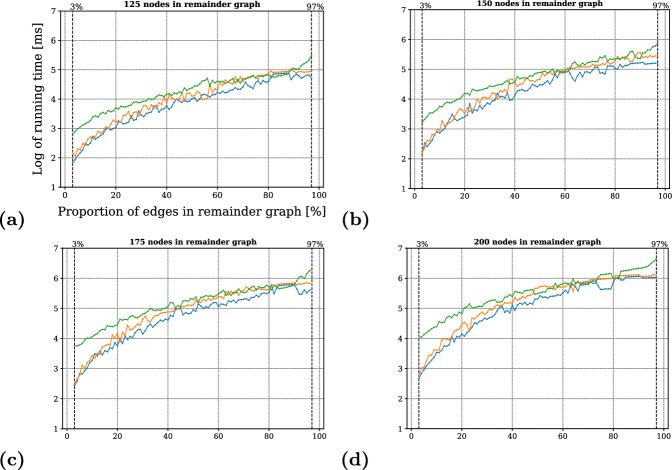
Average running times ($10^k$ [ms] for each $k = 2,..., 7$) for the methods **RB**$_1$ (green), and the custom **RB**$_2$ (orange) and **GM** (blue) over ITS graphs having different number of nodes: **a** 125, **b** 150, **c** 175 and **d** 200 nodes, and having a varying percentage of edges (3% to 97%)

Figure 14 summarizes the distributions of running time of variant **RB-nx** for both **Method A** and **Method B**. We observe outliers explaining the higher values obtained for all three variants under **Method B** with $N_h = 2$. This might be linked, yet again, to reactions whose base ITS graph has a larger automorphism group, with up to a thousand automorphisms, see Fig. 21 a.

**Fig. 18:**
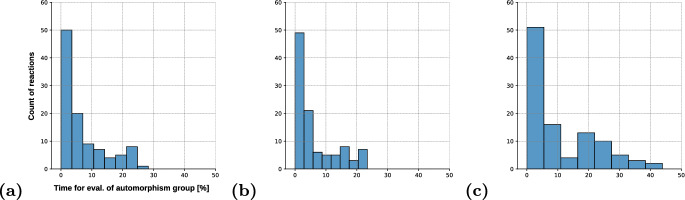
Distributions of time required for obtaining the automorphism group of the base ITS graph used in **Method B**, as a percentage of the total running time of the method over real reactions. Frame **a** shows the distribution for the variant **AN-gm**, **b** for **RB-gm**, and **c** for **RB-nx**. Though **RB-nx** seems to take a greater amount of time for this task, these values are actual explained by the results shown in Table 2, which point to **RB-nx** using less time for the overall analysis for $N_h \ge 3$

**Fig. 21:**
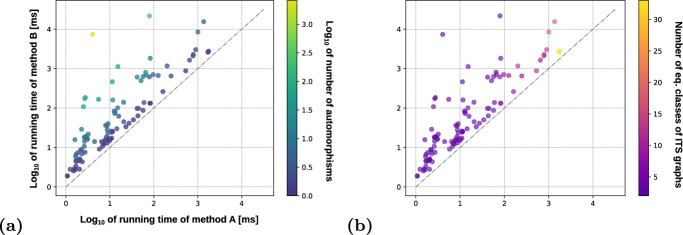
Scatter plots comparing the running times of the variant **AN-gm** for **Method A** in the x-axis and **Method B** in the y-axis, over the data set of real chemical reactions. Through two distinct color gradients, frame **a** shows its relation to the number of automorphisms of the base ITS graph, and frame **b** to the number of equivalence classes of the ITS graphs obtained from the hydrogen extensions of each reaction. The diagonal dotted line is the identity on the real-interval [0, 5], and allows us to see that **Method A** performs better than **Method B**, with respect to both this variant and to this data set

In general, our custom implementations are the variants most sensitive to the size of the isomorphism group and to the interconversion between C++ and Python objects in these functions. Figure 18 in the Appendix shows, however, that the effort for the evaluation is small compared to the effort required for evaluation co-extension of AAMs. These results suggest, thus, that the custom implementations might be better suited for obtaining in only one call the automorphism group in each case, but not for the iterated calls required by the main cycle in Algorithm 4, in order to reduce the costly repeated interconversion of data-types.

*Benchmarking co-extension and HAMP on random graphs*

In order to isolate our analysis from the effects of big automorphism groups, we produced a family of 100 synthetic ITS graphs with the same base AAM and with randomly generated unmatched hydrogen atoms with trivial automorphism group for $N_h=2$, 3, 4, and 5 unmatched hydrogen atoms. The trivial automorphism group group implies that no two distinct hydrogen-extensions are co-extensions. That is, distinct extensions cannot be equivalent AAMs by Definition 3. This implies, in turn, that the clustering of ITS graphs derived from the hydrogen-extensions in our analysis, is reduced automatically to a worst case scenario of exhaustive comparison of unordered pairs of $N_h!$ ITS graphs, and producing at the end $N_h!$ classes with only one representative each, for a reaction with $N_h$ unmatched hydrogens.

This situation is aggravated, furthermore, for the custom variants **AN-gm** and **RB-gm** when used within **Method B**. In addition to the costly interconversion between C++ and Python objects, these variants of *anchored* isomorphism problems also test the consistency of the input anchor against the vertex sets of the input graphs, incurring in further costly Python operations within the already expensive iteration over the unordered pairs of ITS graphs. In contrast, our custom implementations for *direct* isomorphism testing, i.e., those that do not start from an explicit anchor (besides of the relabeling necessary for Algorithm 2), implemented for **Method A** and also available within the GranMapache repository, prove to have the best performance of all the variants as shown in Table 3.

**Table 3:** Average time taken to cluster ITS graphs corresponding to hydrogen extensions of simulated random reactions and number $N_h = 2, 3, 4, 5$ of unmatched hydrogen atoms (mean ± std in ms)

$N_h$	AN-gm	RB-gm	RB-nx
	Method A [ms]		
2	2.05 ± 0.33	2.43 ± 4.13	2.87 ± 0.72
3	17.13 ± 7.44	15.88 ± 0.69	24.95 ± 3.35
4	249.98 ± 8.59	258.49 ± 15.95	396.71 ± 39.14
5	6666.72 ± 244.34	6777.23 ± 259.19	10262.00 ± 987.68
	Method B [ms]		
2	3.90 ± 4.20	3.66 ± 0.41	6.45 ± 4.73
3	25.42 ± 8.12	31.31 ± 12.59	25.64 ± 11.69
4	390.02 ± 21.77	486.39 ± 22.35	312.87 ± 32.57
5	9954.37 ± 226.20	13071.89 ± 299.92	7797.25 ± 600.77

The running time of variant **RB-nx** with **Method B** is shorter than with **Method A** for $N_h = 4, 5$. This indicates that using the automorphism group of a base ITS graph is practically useful for repeated comparison, and in particular for the HAMP. For details we refer to the Appendix.

In order to validate the correct functioning of our implementations, we additionally compared the equivalence classes of hydrogen-extensions obtained with **Method A** and **Method B** for each reaction. In every case both methods identified the same exact classes, i.e., they clustered in the same way the permutations representing the partial map between hydrogen atoms extending the base AAM. This was successfully achieved for both the real chemical reactions, as well as for the synthetic ones. All the scripts used for this analysis can be found inside the GranMapache repository [34].

## Concluding remarks

In this contribution we first gave a comprehensive mathematical description of the relationships between good partial AAMs and full descriptions of the underlying reaction. In particular, we showed that good partial AAMs, i.e., those that cover the reaction center, are exactly the partial AAMs that have unique extensions constituting isomorphisms of the remainder graphs of the reactant and product sides. These results extend our work in [16] by establishing equivalent characterizations of good AAMs. This shows that the practical problem of determining whether a partial AAM is good and, if so, retrieving its unique stable extension, is equivalent to a restricted graph isomorphism problem. Based on these theoretical insights we benchmark different implementations of graph isomorphism tests. Not all such methods lend themselves to incorporate additional constraints implied by the partial AAM. In VF2-like methods, these constitute an immutable set of initial matches. Canonization-based methods can be used after modifying the vertex labels, such that the two vertices of the corresponding pairs $(x,\pi (x))$ are assigned unique matching labels. For comparison we also consider an ILP formulation. Benchmarking simulations show that the completion is feasible for graphs relevant to applications in chemistry. Moreover, we observe that dedicated graph isomorphism algorithms are much more efficient than the ILP.

In contrast to graph-based methods, however, the ILP can potentially be used for *bad* partial AAMs, for which the plausible extensions are, of course, no longer isomorphisms between $\hat{G}_{\pi }$ and $\hat{H}_{\pi }$, and are determined by minimizing the number of necessary reaction edges in an AAM $\alpha$ extending $\pi$ [16]. This is of practical importance since most reaction mapping tools do not predict correspondences between hydrogen atoms, though these often take part in the reactions and hence are reacting vertices.

Provided the hydrogen atoms taking part in the reaction can be identified, Theorem 8 shows that isomorphisms between ITS graphs that lack hydrogens can be extended to the representation including hydrogens. More generally, it is sufficient that not all isomorphisms map vertices that are only present in the extended graph to atoms already present in the reduced representation, i.e., at least one isomorphism should stabilize the reduce representation. This condition is of course always satisfied for implicit and explicit hydrogens atoms, but can be violated e.g. by other types of data with bad partial AAMs, such as the KEGG RCLASSes [15]. Bad partial AAMs also arise from unbalanced reaction data. These convey an even more difficult problem, in particular if both the reactant and the product sides of the reaction are incompletely specified. The problem of efficient comparisons between such data remains an open problem for future research.

## Figures and Tables

**Fig. 19:**
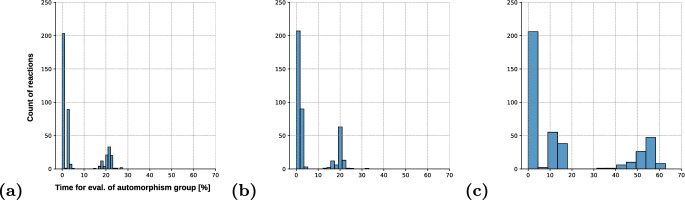
Analogous results to Fig. 18 for the **Method B**, now over the simulated random reactions. Again **a** shows the distribution for the variant **AN-gm**, **b** for **RB-gm**, and **c** for **RB-nx**. Similarly to the analysis over real reactions, in this case Table 3 suggest that **RB-nx** uses less time for the overall analysis for $N_h \ge 3$, explaining an apparent higher percentage of time spent for evaluating the automorphism group

**Fig. 20:**
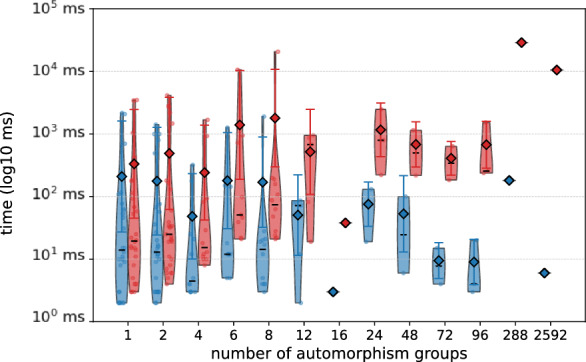
Benchmarking of hydrogen–extension comparison for **method A** (blue) and **method B** (red) over the set of 104 real chemical reactions, both implemented with the custom function for anchored isomorphism search from the GranMapache repository [34]. The running times are grouped by the cardinality of the automorphism group of the base ITS graph. Both methods become slower for highly symmetric ITS graphs

**Fig. 22:**
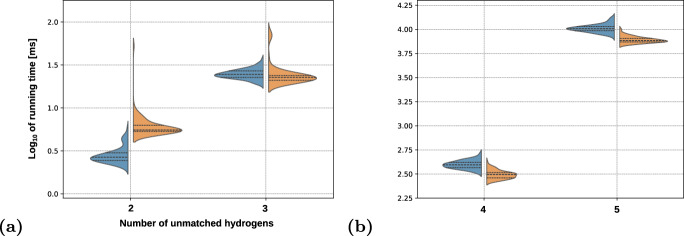
Mirrored distributions of the running times of **RB-nx**, with respect again to **Method A** in blue, and **Method B** in orange. Here we exhibit the results of the analysis over simulated random reactions. To better appreciate each distribution we split them into two separate plots with the same scale but different range of values. Frame **a** covers the results for $N_h = 2, 3$ unmatched hydrogens and **b** covers the cases $N_h = 4, 5$. Quartiles are indicated by the horizontal dotted lines

## Data Availability

All used data sets and custom code implementations are available in the following public-access repositories: https://github.com/TieuLongPhan/PartialAAMshttps://github.com/MarcosLaffitte/GranMapachehttps://github.com/MarcosLaffitte/GranMapache/tree/main/examples/Stable_Extensionshttps://github.com/MarcosLaffitte/GranMapache/tree/main/examples/Hydrogen_Extensions The experimental repository has also been permanently archived on Zenodo under DOI 10.5281/zenodo.17649864 and can be directly installed via pip install partialaam.

## Appendix A appendix: additional figures and tables

### Further performance data of **RB-nx**

The use of the **RB-nx** variant with **Method B** has some limitations visible for the case $N_h = 2$ in Table 3 and Fig. 22. Here, the comparison of only one pair of ITS graphs through the (only) automorphism of the base ITS graph, turns out to be slower than their straightforward comparison. This is expected, however, since from a theoretical point of view such use of Algorithm 4 would be approximately equal to carrying the same search twice. This suggests, nevertheless, that the benefits of using this specific variant, fully dependent on Python, might not outweigh the benefits when comparing a small number of hydrogen-extensions (Figs. 19, 20).

## Declarations

### Editorial Policies for:

Springer journals and proceedings: https://www.springer.com/gp/editorial-policies

### Nature Portfolio journals:

https://www.nature.com/nature-research/editorial-policies

### *Scientific Reports*:

https://www.nature.com/srep/journal-policies/editorial-policies

### BMC journals:

https://www.biomedcentral.com/getpublished/editorial-policies
